# Interneuron Dysfunction in a New Mouse Model of SCN1A GEFS+

**DOI:** 10.1523/ENEURO.0394-20.2021

**Published:** 2021-04-08

**Authors:** Antara Das, Bingyao Zhu, Yunyao Xie, Lisha Zeng, An T. Pham, Jonathan C. Neumann, Olga Safrina, Daniel R. Benavides, Grant R. MacGregor, Soleil S. Schutte, Robert F. Hunt, Diane K. O’Dowd

**Affiliations:** 1 Department of Developmental and Cell Biology, University of California, Irvine, CA 92697; 2 Department of Anatomy and Neurobiology, University of California, Irvine, CA 92697; 3 Transgenic Mouse Facility, Office of Research, University of California, Irvine, CA 92697

**Keywords:** CRISPR/Cas9, epilepsy, GEFS+, parvalbumin interneurons, SCN1A, seizures

## Abstract

Advances in genome sequencing have identified over 1300 mutations in the *SCN1A* sodium channel gene that result in genetic epilepsies. However, it still remains unclear how most individual mutations within *SCN1A* result in seizures. A previous study has shown that the K1270T (KT) mutation, linked to genetic epilepsy with febrile seizure plus (GEFS+) in humans, causes heat-induced seizure activity associated with a temperature-dependent decrease in GABAergic neuron excitability in a *Drosophila* knock-in model. To examine the behavioral and cellular effects of this mutation in mammals, we introduced the equivalent KT mutation into the mouse (*Mus musculus*) *Scn1a* (*Scn1a^KT^)* gene using CRISPR/Cas9 and generated mutant lines in two widely used genetic backgrounds: C57BL/6NJ and 129X1/SvJ. In both backgrounds, mice homozygous for the KT mutation had spontaneous seizures and died by postnatal day (P)23. There was no difference in mortality of heterozygous KT mice compared with wild-type littermates up to six months old. Heterozygous mutants exhibited heat-induced seizures at ∼42°C, a temperature that did not induce seizures in wild-type littermates. In acute hippocampal slices at permissive temperatures, current-clamp recordings revealed a significantly depolarized shift in action potential threshold and reduced action potential amplitude in parvalbumin (PV)-expressing inhibitory CA1 interneurons in *Scn1a^KT/+^* mice. There was no change in the firing properties of excitatory CA1 pyramidal neurons. These results suggest that a constitutive decrease in inhibitory interneuron excitability contributes to the seizure phenotype in the mouse model.

## Significance Statement

A fundamental challenge in understanding the etiology and developing treatments for genetic epilepsies is the heterogeneity of the phenotypes. Over 1300 genetic mutations in *SCN1A*, a gene that encodes the voltage gated sodium channel Nav1.1, are associated with epilepsies of widely different severity. The mechanisms by which the R1648H *SCN1A* mutation result in a genetic epilepsy with febrile seizure plus (GEFS+) phenotype have been studied extensively in a well-established knock-in mouse model. To understand how other GEFS+ causing *SCN1A* mutations affect neural circuits and behaviors, we established a second knock-in mouse model using CRISPR/Cas9 editing. The K1270T *SCN1A* knock-in mice represent an important tool for identifying similarities and differences in mechanism of action with R1648H and other epilepsy mutations, and for development of mutation-specific therapies.

## Introduction

Mutations in the *SCN1A* gene, which encodes the α subunit of the voltage-gated sodium channel Nav1.1, result in a broad spectrum of genetic epilepsies. Among 1300 known epilepsy causing *SCN1A* mutations, ∼10% of the missense mutations are associated with genetic epilepsy with febrile seizures plus (GEFS+; Zhang et al., 2017; Miller and Sotero de Menezes, 2019). Individuals with GEFS+ exhibit seizures induced by fever (febrile seizures) that begin in infancy but persist beyond six years of age (hence, febrile seizures plus; FS+). Some GEFS+ patients develop additional seizure phenotypes later in life, including afebrile generalized tonic-clonic, myoclonic or absence seizures (Scheffer, 1997; Abou-Khalil et al., 2001; Escayg et al., 2001; Meng et al., 2015; Schutte et al., 2016; Zhang et al., 2017). The mechanisms underlying the GEFS+ disorder associated with different missense mutations remain poorly understood. Moreover, variations in seizure severity can occur even in family members with identical *SCN1A* mutations (Abou-Khalil et al., 2001; Zhang et al., 2017), suggesting that environmental factors and/or genetic modifiers can affect the phenotype (Bergren et al., 2009; Hawkins et al., 2011; Jorge et al., 2011; Hawkins and Kearney, 2016). This has made developing effective therapies for treating GEFS+ patients challenging.

Genetic models with specific missense mutations have been important in evaluating the cellular and circuit mechanisms contributing to GEFS+. A rat model of GEFS+ (SCN1A N1417H) demonstrated reduced action potential amplitudes in hippocampal interneurons that are likely to contribute to the hyperthermia-induced seizure phenotype (Mashimo et al., 2010; Hayashi et al., 2011). A genetic knock-in model for *SCN1A* epilepsy in mice carrying the GEFS+ causing R1648H mutation, results in animals that exhibit heat-induced seizures as well as sleep, cognitive and social behavior deficits. Evaluation of the electrophysiological properties of hippocampal and cortical neurons in brain slices or dissociated neurons reveals impaired action potential firing in inhibitory neurons, consistent with the idea that alterations in the excitation/inhibition (E/I) balance contribute to heat-induced seizures (Martin et al., 2010; Hedrich et al., 2014). Given the large number of *SCN1A* mutations that give rise to genetic epilepsy, mouse models with mutations other than R1648H are critical for identifying common as well as distinct mechanisms by which single mutations can result in a GEFS+ phenotype. This will ultimately facilitate the development of personalized anti-epileptic medicine.

In this study, we focused on the K1270T mutation identified in a large, multigenerational family. A previous study on a GEFS+ K1270T knock-in *Drosophila* line, carrying the mutation in the fly’s only sodium channel gene *para*, resulted in alterations in sodium currents at high temperatures that reduced firing specifically in inhibitory neurons, contributing to a heat-induced seizure phenotype (Sun et al., 2012). Another study, evaluating the K1270T mutation in human induced pluripotent stem cell (hiPSC)-derived neurons also revealed reduced excitability of inhibitory neuron but the underlying alterations in sodium currents appear to be different in the fly and the stem cell model (Xie et al., 2020). Here, we used CRISPR/Cas9 to generate a new mouse model of the GEFS+ *SCN1A* K1270T mutation. This new knock-in mouse model allows additional comparison of the effect of the K1270T mutation between species, and in conjunction with the R1648H mouse model broadens our ability to explore how distinct missense mutations alter cellular and circuit mechanisms underlying *SCN1A* GEFS+ phenotypes.

## Materials and Methods

### Animals

Mice were maintained on a 12/12 h light/dark cycle [lights on: zeitgeber time (ZT)0 at 6:30 A.M. local time and lights off: ZT12 at 6:30 P.M. local time] with *ad libitum* food (Envigo-Teklad global rodent diet #2020×) and water. Mice were housed in groups and not individually. All animal procedures were performed in accordance with the University of California, Irvine Institutional Animal Care and Use Committee's regulations. Scn1a*K1259T mutant mice (hereafter referred to as *Scn1a^KT^*) modeling human SCN1A*K1270T were generated by CRISPR/Cas9 and maintained on a C57BL/6NJ (JAX stock #005304) or 129X 1/SvJ (JAX stock #000691) background. Details of the targeting of *Scn1a* are provided below. In some experiments, *Scn1a^KT/+^* mice were mated with a PV-Cre (JAX stock #017320) and Ai14-td-Tomato reporter (JAX stock #007908) on a C57BL/6J background to generate wild-type and heterozygous mice that have parvalbumin (PV) neurons endogenously labeled with fluorescent red td-Tomato marker.

### Generation of *Scn1a^KT^* mice

Mutant mice were generated by introducing the human K1270T missense mutation within exon 19 at an equivalent position (K1259T) in mouse *Scn1a* gene via CRISPR/Cas9 in combination with a single-stranded oligodeoxynucleotide (ssODN) for use in homology-dependent repair (HDR). PCR was used to synthesize a DNA template that was subsequently used to produce the sgRNA72 using T7 RNA polymerase. The DNA template was generated by PCR using a forward primer (5′-gttaatacgactcactatagTCCTGGAGATGCTCCTCAAAgtttaagagctatgctg-3′) that contained (from 5′ to 3′) a T7 ribopromoter, the 20-base guide sequence (TCCTGGAGATGCTCCTCAAA), and a region of homology to plasmid pSLQ1651-sgTelomere(F + E) (Addgene #51024; Chen et al., 2013). The reverse primer (5′-GAAAAAAAGCACCGACTCGGTGCC-3′) defined the 3′ end of the single guide RNA (sgRNA72). Thirty cycles of PCR was performed using both primers, 5 ng of pSLQ1651-sgTelomere(F + E) and 1 μm each primer plus 200 μm dNTPs. After purification of the PCR product, T7 RNA polymerase was used to synthesize the sgRNA (sgRNA72). Messenger RNA encoding Cas9 was synthesized using T7-mediated *in vitro* transcription of bbCas9pluspAAA (Addgene #82581; Pritchard et al., 2017) followed by capping and poly-adenylylation as directed by the manufacturer (mMessage mMachine T7 ultra transcription kit, Thermo Fisher). B6SJLF2/J fertilized mouse oocytes were microinjected with sgRNA72 (10 ng/μl), Cas9 mRNA (10 ng/μl) and a modified single-stranded deoxyribonucleotide (ssODN; Integrated DNA Technologies, Ultramer) as a template for HDR. The ssODN contained the desired sequence to introduce the K1259T amino acid change, silent base changes to reduce likelihood of re-cutting of the desired allele, and a silent base change to generate an EcoRV site for screening (5′-ACCATGCTGGAGTATGCTGACAAAGTCTTCACTTACATTTTCATCCTtGAGATGCTCCTgAccTGGGTcGCCTATGGaTATCAAACATACTTCACCAATGCCTGGTGTTGGCTAGACTTCTTA-3′). Surviving oocytes were transferred to the reproductive tract of pseudo-pregnant CD-1 females. After screening of offspring, a single male with the desired modification (#1472) was used to establish two lines of *Scn1a*^KT^ mice: one on a C57BL/6NJ and a second on a 129X1/SvJ strain background by backcrossing with wild-type female mice (JAX stock #005304 or #000691, respectively) for at least five generations.

### PCR

To screen for mice with the *Scn1a^KT^* mutation, DNA extracted from toe biopsies (lysis buffer and proteinase K, Viagen) was amplified using a *Scn1a*-specific forward primer (5′-TTCCATCCCAAGGAAATACCATGT-3′) and a reverse primer (5′-GCCTATCTTGTCATCACAACACAGTG-3′). PCR amplification was performed for 35 cycles of 94°C for 30 s, 59°C for 30 s, and 72°C for 30 s using recombinant 5 U/μl Taq polymerase was prepared in 10× buffer with 50 mm MgCl_2_ (Invitrogen) in the reaction mix. The 388-bp PCR amplicon was digested with 20 U EcoRV restriction enzyme prepared in 10× NEB 3.1 buffer (New England Biolabs) and incubated at 37°C for 60 min to distinguish between the wild-type (388 bp) and mutant (223 and 165 bp) alleles.

### Off-target screening

Potential off-target effects for CRISPR/Cas9 + sgRNA72 were screened using online tools Cas-OFFinder (http://www.rgenome.net/cas-offinder/) and Synthego CRISPR design tool (https://www.synthego.com/products/bioinformatics/crispr-design-tool). Genomic DNA was extracted from the mouse tail biopsies with DirectPCR lysis reagent (Viagen Biotech) and proteinase K (Roche). Amplicons were produced with Taq polymerase with the following conditions: 95°C for 2 min (95°C for 30 s + 60°C for 30 s + 72°C for 30 s) × 35 cycles, and 72°C for 7 min. PCR products were confirmed by gel electrophoresis, purified (QIAquick PCR Purification kit, QIAGEN), subjected to Sanger sequencing (Retrogen) and analyzed in Mac Vector software. Primers for PCR amplification and sequencing for off-target analysis are listed in Extended Data Table 1-1.

### Heat-induced seizures

Wild-type and *Scn1a^KT/+^*mice, postnatal day (P)30–P40 of both sexes and weighing ≥15 g, were used to screen for occurrence of heat-induced behavioral seizures. Each mouse was anesthetized using 1–2% isoflurane for 30–45 s and a rectal temperature probe (RET 3, Braintree Scientific) was inserted and secured to the tail to monitor body temperature throughout the experiment. After anesthesia, animals were allowed to recover in their home cages for at least 15 min during which time the animals resumed normal activity and their core body temperature stabilized at 36–37°C. Animals were introduced into a custom-built forced air chamber fitted with a digital temperature controller and a thermocouple device to control and maintain the set temperature. A Plexiglas front panel enabled video recording of each heating episode. Seizures were scored offline by an experimenter blind to mouse genotype, based on a modified Racine scale: 1, mouth and facial movements; 2, head nodding; 3, forelimb clonus, usually one limb; 4, forelimb clonus with rearing; and 5, generalized tonic-clonic seizure, rearing, falling over (Cheah et al., 2012).

For each animal, the seizure threshold temperature, latency to seizure and seizure behavior score were measured. Seizure threshold was defined as the temperature at which the first behavioral seizure of any severity was observed. Seizure latency was defined as the time (min) from the introduction of the mouse into the heating chamber (time = 0) to the onset of the first behavioral seizure at seizure threshold as described above. The experiment was terminated when (1) the mouse completed a seizure bout, or (2) body temperature of the mouse reached 44°C. Following retrieval from the heat chamber, each mouse was placed on a prechilled dish to rapidly restore normal body temperature. Of the 46 mice that underwent the heat-induced assay, ∼15% of mice (six on 129X1 and one on B6N) did not recover from heat-induced seizures. The remaining 39 mice were returned to home cage after they resumed normal activity and grooming. All mice used for the experiment were euthanized at the end of the day. All experiments were done between ZT3 and ZT10, where ZT0 is 6:30 A.M. local time.

### EEG monitoring

EEG traces were obtained in wild-type and heterozygous mice between three and four months of age (Pinnacle Technologies) as previously described (Kim et al., 2018). Each mouse was anesthetized with ketamine and xylazine (10 and 1 mg/kg, i.p.) until there was no limb-withdrawal response to noxious foot pinch. Prefabricated EEG headsets (#8201, Pinnacle Technologies) were surgically implanted overlying the hippocampus, and cemented in place with a fast-acting adhesive and dental acrylic. Two wires were placed on the shoulder muscles for electromyographic (EMG) recording. Mice were allowed to recover for 5–7 d before recordings were initiated. After recovery from surgery, each mouse was placed in a cylindrical Plexiglas chamber for continuous EEG monitoring over 7–9 d (24 h d^−1^). Electrographic EEG seizures episodes were manually detected by scanning the raw data files using Sirenia Seizure software (Pinnacle Technologies) while the experimenter was blind to mouse genotype. Electrographic seizures were defined as high-frequency, high-voltage synchronized polyspike waves with amplitudes at least twofold greater than background that lasted ≥15 s.

### Cell quantification

Wild-type and heterozygous mice with genetically labeled PV neurons (*Scn1a^+/+^; PV-Cre; Ai14-td-Tomato* or *Scn1a^KT/+;^ PV-Cre; Ai14-td-Tomato*) were transcardially perfused with 4% paraformaldehyde (PFA). Brains were harvested and postfixed in 4% PFA at 4°C overnight and processed as described previously (Frankowski et al., 2019). Coronal serial sections (50 μm) of the brain were cut and mounted on charged slides (Superfrost plus, Fisher Scientific) in Fluoromount G (Southern Biotech) medium. Sections were imaged using Zeiss Axio Imager.M2 fluorescence microscope with a 10× objective and cell counts were performed with ZEN software. Td-Tomato-labeled PV cells were counted in both hemispheres from three brain slices per animal from the dorsal hippocampus in the CA1 region. In each brain slice, PV cell counts were normalized to the area of the CA1 to obtain PV cell density (cells/mm^2^). PV cell density counted across 18 brain sections, across both hemispheres, obtained from three mice/genotype were averaged to determine mean cell density (cells/mm^2^) for each genotype. Mean PV cell density between wild-type and heterozygous mice (*n* = 3) was compared using two-tailed unpaired Student’s *t* test.

### Whole-cell electrophysiology

Electrophysiological recordings were obtained from excitatory pyramidal cells and td-Tomato labeled PV inhibitory interneurons from the CA1 region of the hippocampus. Brains were quickly harvested from deeply anesthetized wild-type and *Scn1a^KT/+^*C57BL/6NJ littermates (P28–P40), and 300- to 350-μm coronal brain slices were prepared in oxygenated ice-cold high-sucrose artificial CSF (ACSF) containing: 150 mm sucrose, 124 mm NaCl, 3 mm KCl, 1.25 mm NaH_2_PO_4·_H_2_O, 2 mm MgSO_4·_7H_2_O, 26 mm NaHCO_3_, 10 mm dextrose, and 2 mm CaCl_2_. Slices were kept at 32°C for at least 30 min before electrophysiological recordings using Axopatch 700B, Digidata 1440A, and pCLAMP10.1 or 10.2 software. Electrophysiological recordings were performed at room temperature (25°C) with glass pipettes with open tip resistance of 3–6 MΩ (TW150F-3, World Precision Instruments or #534332-921 100-μl calibrated pipettes, VWR International). The bath solution consisted of ACSF: 124 mm NaCl, 3 mm KCl, 1.25 mm NaH_2_PO_4·_H_2_O, 2 mm MgSO_4·_7H_2_O, 26 mm NaHCO_3_, 10 mm dextrose, and 2 mm CaCl_2_. Electrodes were filled with internal solution either (1) 120 mm potassium gluconate, 20 mm NaCl, 0.1 mm CaCl_2_, 2 mm MgCl_2_, 1.1 mm EGTA; 10 mm HEPES, 4.5 mm Na_2_ATP (pH 7.2); or (2) 140 mm potassium gluconate, 1 mm NaCl, 1 mm CaCl_2_, 1 mm MgCl_2_, 5 mm EGTA, 10 mm HEPES, 3 mm KOH, and 2 mm ATP (pH 7.2). Osmolarity of external ACSF and internal solution was adjusted to 310–312 and 288–290 mOsm, respectively, using VAPRO 5200 osmometer (Wescor). Signals were obtained using pCLAMP10.2 with a Bessel filter at 2–4 kHz and a low-pass filter at 10 kHz. The passive properties of the cell were determined in voltage clamp mode. Resting membrane potentials were measured immediately after break-in with no current injection at current clamp mode. To record firing properties, cells were injected with a series of long (1000-ms) current steps in 10-pA increments, between –80 and 200 pA for excitatory neurons and –80 and 900 pA for PV inhibitory neurons. The number of action potentials fired in 1000 ms was compared between wild-type and heterozygous mice. Data analysis was performed using pCLAMP Clampfit10.6 and Microsoft Excel.

### Quantification and statistics

All datasets were analyzed with D’Agostino and Pearson normality test before carrying out parametric statistical tests; datasets that did not follow a normal distribution were compared using nonparametric Mann–Whitney test. Survivorship curves were compared between wild-type, heterozygous, and homozygous mice with log-rank Mantel–Cox test. Mouse mean body weights for each genotype were calculated with at least three mice per time point. Mean body weight across each day was compared among homozygous, heterozygous, and wild-type mice with multiple *t* tests of two-way ANOVA. Body weights between heterozygous and wild-type mice in respective strain background were compared using two-way ANOVA with genotype and postnatal age (days) as factors, followed by Sidak’s *post hoc* multiple comparisons. Rate of change of body temperature across time, seizure threshold temperature, seizure latency and Racine score were compared between wild-type and heterozygous mice using two-tailed unpaired Student’s *t* test or Mann–Whitney test as specified in the text. Percentage of mice that had heat-induced seizures was compared between genotypes with Fisher’s exact test.

Mean PV cell density between wild-type and heterozygous mice was compared using two-tailed unpaired Student’s *t* test. For each individual recorded cell, the action potential (AP) threshold, amplitude, and half-width were measured from the first evoked action potential. Phase graphs were generated by plotting the first derivative (dv/dt) of the AP waveform against the membrane potential of the AP trace (Vm). AP threshold was calculated as the voltage at which the first derivative (dV/dt) of the AP waveform reached 10 mV/ms (Favero et al., 2018). AP amplitude was computed as the height from AP threshold (determined from phase plots) to the maximum peak of the AP waveform. Action potential characteristics and passive cell membrane properties (cell capacitance, input resistance and resting membrane potential) were compared between genotypes using two-tailed unpaired Student’s *t* test or Mann–Whitney test as specified in the text (*p *<* *0.05). Curves of firing frequency versus injected currents were compared between heterozygous and wild-type mice using two-way ANOVA (genotype and injected current as factors) with repeated measures. To determine spike frequency adaptation, ratio of interspike interval (ISI) between the first two and last two spikes (ISI_1_/ISI_n_) was calculated in Clampfit and compared between genotypes using Mann–Whitney test. ISI duration was determined from the first suprathreshold current injection step that elicited sustained tonic firing, as described in a previous study (Favero et al., 2018). All statistical tests were performed in Prism 8 (Graph Pad Software Inc.). Representative neuronal firing traces were plotted using MATLAB (MathWorks), all other data figures were plotted using Prism 8 (Graph Pad Software Inc.) and Delta Graph 7.1 (RedRock Software).

## Results

### Generation of *Scn1a^KT^* mutant mice

**Figure 1.**
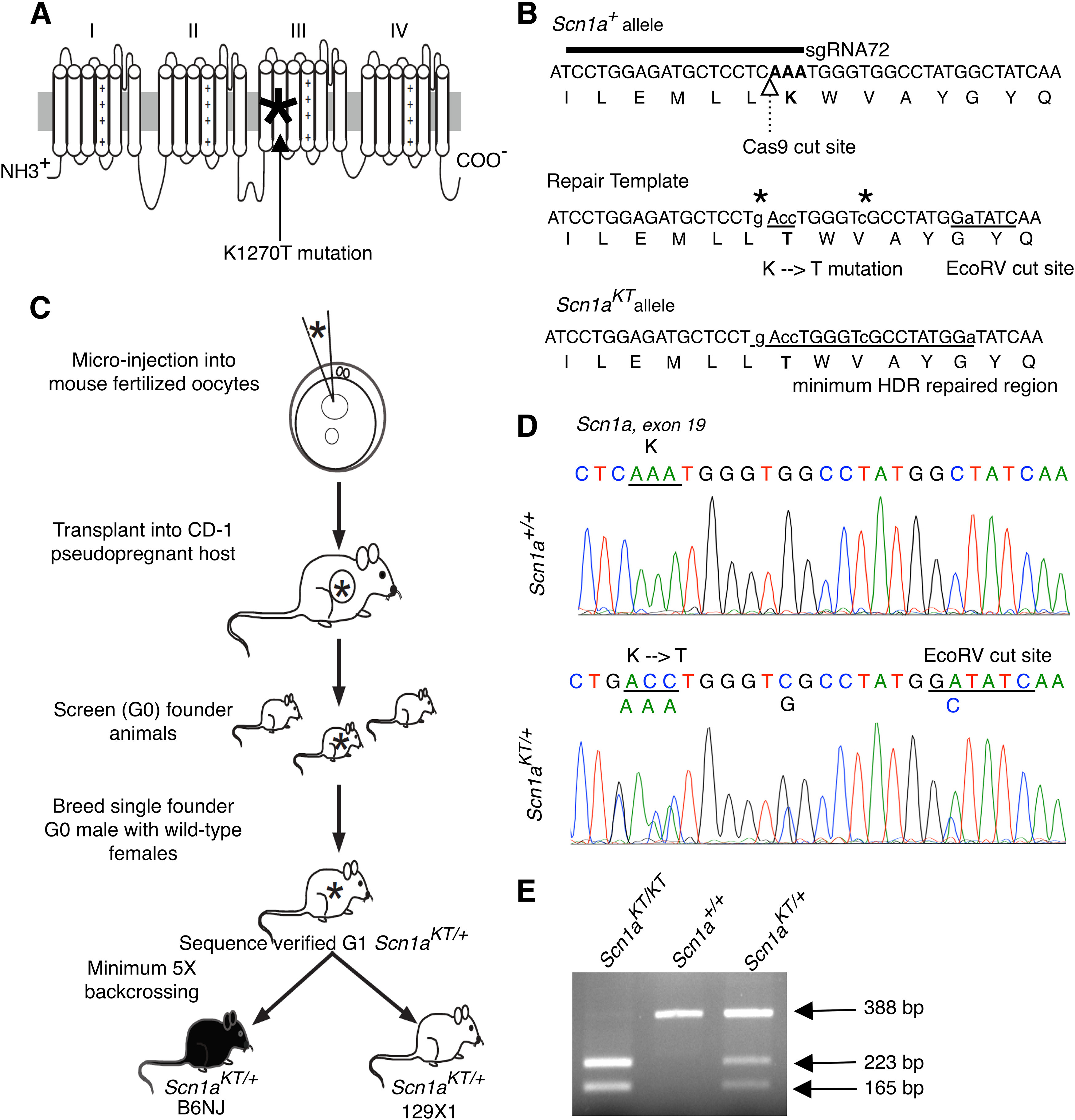
Generation of *Scn1a^KT/+^* mouse using CRISPR/Cas9. ***A***, The GEFS+ causing K1270T mutation (asterisk) is located in S2 transmembrane segment of Domain III of the α subunit of Nav1.1 ion channel encoded by the human *SCN1A* gene. ***B***, Location of the guide RNA relative to the Cas9 cut site and the locus of the K1259T mutation in the mouse *Scn1a* gene and the Nav1.1 protein sequence. Repair template sequence with the base pair changes introducing the K-T mutation and the EcoRV cut site. All edited nucleotides are shown in lower case letters and the HDR region is represented by underlined letters. Two additional silent mutations (asterisks) were added to prevent re-cutting by Cas9 following HDR. ***C***, Outline of the steps followed to generate *Scn1a^KT^* mouse colonies in B6NJ and 129X1 genetic backgrounds from a single founder G_0_ male. ***D***, DNA sequence comparison between a wild-type (*Scn1a^+/+^*) and a heterozygous (*Scn1a^KT/+^*) mouse showing missense K-T mutation and another silent mutation that results in an EcoRV cut site. DNA chromatograms of *Scn1a^+/+^*and *Scn1a^KT/+^* mice showing no off-target effects is shown in Extended Data Figure 1-1. ***E***, A representative agarose gel shows PCR amplified DNA bands digested with EcoRV which distinguishes between mice homozygous for the mutant allele, *Scn1a^KT/KT^* (223 and 165 bp), *Scn1a^+/+^* wild-type mice homozygous for the wild-type allele (388 bp), and heterozygous *Scn1a^KT/+^* mice carrying one copy each of wild-type (388 bp) and mutant (223 and 165 bp) alleles.  *Figure Contributions:* Grant R. MacGregor and Jonathan C. Neumann designed the CRISPR/Cas9 strategy to generate transgenic mouse. Soleil S. Schutte, Antara Das performed DNA sequencing and genotyping. Grant MacGregor, Olga Safrina, and Daniel R. Benavides performed off-target screening, Antara Das and Olga Safrina, analyzed the off-target data.

The human GEFS+ associated K1270T mutation is located in the second transmembrane region of the third homology domain of the Nav1.1 sodium channel (Fig. 1*A*, asterisk). The equivalent amino acid in the mouse Nav1.1 channel is at K1259 (Fig. 1*B*). To generate a *SCN1A* K1270T missense mouse model, a single guide RNA (sgRNA72; Fig. 1*B*) was used (see Materials and Methods) to target the Cas9 cut site immediately upstream of the desired KT mutation in exon 19 (c.4227A) of the mouse *Scn1a* gene (Fig. 1*B*). The repair template introduced a two-base pair mutation that converts a lysine (K: AAA) residue to threonine (T: ACC). For initial screening and routine genotyping, a silent mutation that results in an EcoRV restriction site was also included in the repair template (Fig. 1*B*). The DNA sequence modified by HDR is tracked by incorporation of silent mutations introduced via the repair template (Fig. 1*B*). Following CRISPR mediated gene editing, the *Scn1a^KT^* allele carries the desired missense mutation that results in a K to T conversion, an EcoRV restriction enzyme cut site, and two additional silent mutations to prevent Cas9 activity on the repaired allele. To remain consistent with the nomenclature in human subjects (Abou-Khalil et al., 2001), the mutant allele is referred to as *Scn1a^KT^* (K1270T; Fig. 1*B*). Single guide RNA, plus Cas9 mRNA and ssODN repair template were injected into mouse zygotes, and surviving embryos were transplanted into pseudo-pregnant CD-1 females (Fig. 1*C*). A single founder G_0_ male was identified that contained the *Scn1a^KT^* mutation and the EcoRV site, confirmed by PCR amplification and sequencing. To establish stable breeding lines in two different genetic backgrounds, this founder male was bred to C57BL/6NJ (or B6NJ) and 129X1/SvJ (or 129X1) females. Lines were backcrossed for at least five generations (Fig. 1*C*). Transmission of the mutant allele was verified by PCR amplification and sequencing to confirm the presence of both the *Scn1a^KT^* mutation and EcoRV site at the appropriate locations in the offspring in subsequent generations (Fig. 1*D*). Routine genotyping of animals took advantage of the EcoRV site to distinguish between wild-type, heterozygous, and homozygous mutant animals (Fig. 1*E*). Restriction digestion of the 388-bp PCR product from the mutant allele by EcoRV produced 223 and 165 bp products, while the wild-type allele 388 bp product is not digested.

To determine whether the mutant lines contained confounding off-target mutations that can arise during CRISPR gene editing we identified potential off-targets sites using Cas-OFFinder (Bae et al., 2014) and the Synthego CRISPR design tool. Since both *Scn1a^KT/+^* mutant lines were backcrossed to wild-type B6NJ or 129X1 backgrounds for a minimum of five generations, it was unlikely that a mutation caused by an off-target effect of CRISPR/Cas9 would be present on a chromosome other than chromosome 2, the location of *Scn1a* (C57BL/6NJ; Chr2; 66.3 Mb, GRCm38.p6, Ensembl release 98). Therefore, the analysis focused on potential off-target sites on chromosome 2. Three sites were identified containing up to three mismatches (Table 1). One site was in *Scn3a* and a second in *Scn7a*; both are paralogs of *Scn1a* located within 1 Mb of *Scn1a*. The third site was in *Cd44* gene, located at 102.8 Mb, that encodes a multifunctional cell-surface glycoprotein involved in cell-cell interaction, adhesion, and migration (Ruiz et al., 1995; Isacke and Yarwood, 2002). DNA from two wild-type *Scn1a^+/+^* and two heterozygous *Scn1a^KT/+^* mice were amplified by PCR (Extended Data Table 1-1) and sequenced across the potential off-target region at each locus. There was no evidence of off-target mutations based on the homozygous wild-type genomic DNA sequence observed at the three top potential off-target sites in all animals (Table 1; Extended Data Fig. 1-1).

**Table 1:** Summary of off-target analysis

sgRNA72 crRNA TCCTGGAGATGCTCCTCAAA					
Genomic DNA	Chr	Position	Mismatches	Locus	Off-target effect
TCCTGGAGATGCTCCTCAAA|TGG	chr2	66299689	0	*Scn1a*	N/A
TCCTGGA**a**ATGCTCCTCAAA|TGG	chr2	65482216	1	*Scn3a*	No
T**t**CTGGAGATGCT**t**CTCAA**g**|TGG	chr2	66689597	3	*Scn7a*	No
T**t**CTGGAGATGCTCCTCA**tt**|CGG	chr2	102858300	3	*Cd44*	No

N/A, not applicable. Results of Cas-OFFinder analysis of sgRNA72 targets on mouse chromosome 2. The intended target for sgRNA72 is within *Scn1a*, shown in green. The location of each of the three potential off-target sites on mouse chromosome 2 identified by Cas-OFFinder is shown. The mismatches are indicated by lower case bold letters. The underlined region indicates the 10-nucleotide seed region proximal to the PAM site. The vertical line at the 3′ end of each target or off-target site indicates the boundary between the gRNA target and the adjacent Cas9 PAM (NGG) sequence. No off-target effects were found when *Scn1a* K1270T WT and heterozygous mouse DNA was amplified using specific primers (shown in Extended Data Table 1-1) and sequences were analyzed (Extended Data Fig. 1-1).

10.1523/ENEURO.0394-20.2021.tab1-1 Extended Data Table 1-1 Primers for PCR amplification and sequencing for off-target analysis. The table lists the genes, exon positions, and sequences of the forward and reverse primers used to screen off-target effects in *Scn1a* K1270T mice. Fwd, forward; Rev, reverse. Download Table 1-1, DOCX file.

10.1523/ENEURO.0394-20.2021.f1-1 Extended Data Figure 1-1 Analysis of off-target effects. Chromatograms of DNA sequence of two wild-type *Scn1a^+/+^* (#17 and #26) and two heterozygous *Scn1a ^KT/+^* (#21 and #23) mice, obtained from different parents are shown. No off-target effects were detected at three loci on mouse chromosome 2, namely, *Scn3a*, *Scn7a*, and *Cd44*. At each locus, the region highlighted in blue corresponds to the potential off-target site for the guide RNA (sgRNA72) and the red line denotes the PAM sequence. The Cas9 cut site is shown by the vertical double arrowhead line. Download Figure 1-1, DOCX file.

### *Scn1a^KT/KT^* mice have reduced lifespan

**Figure 2.**
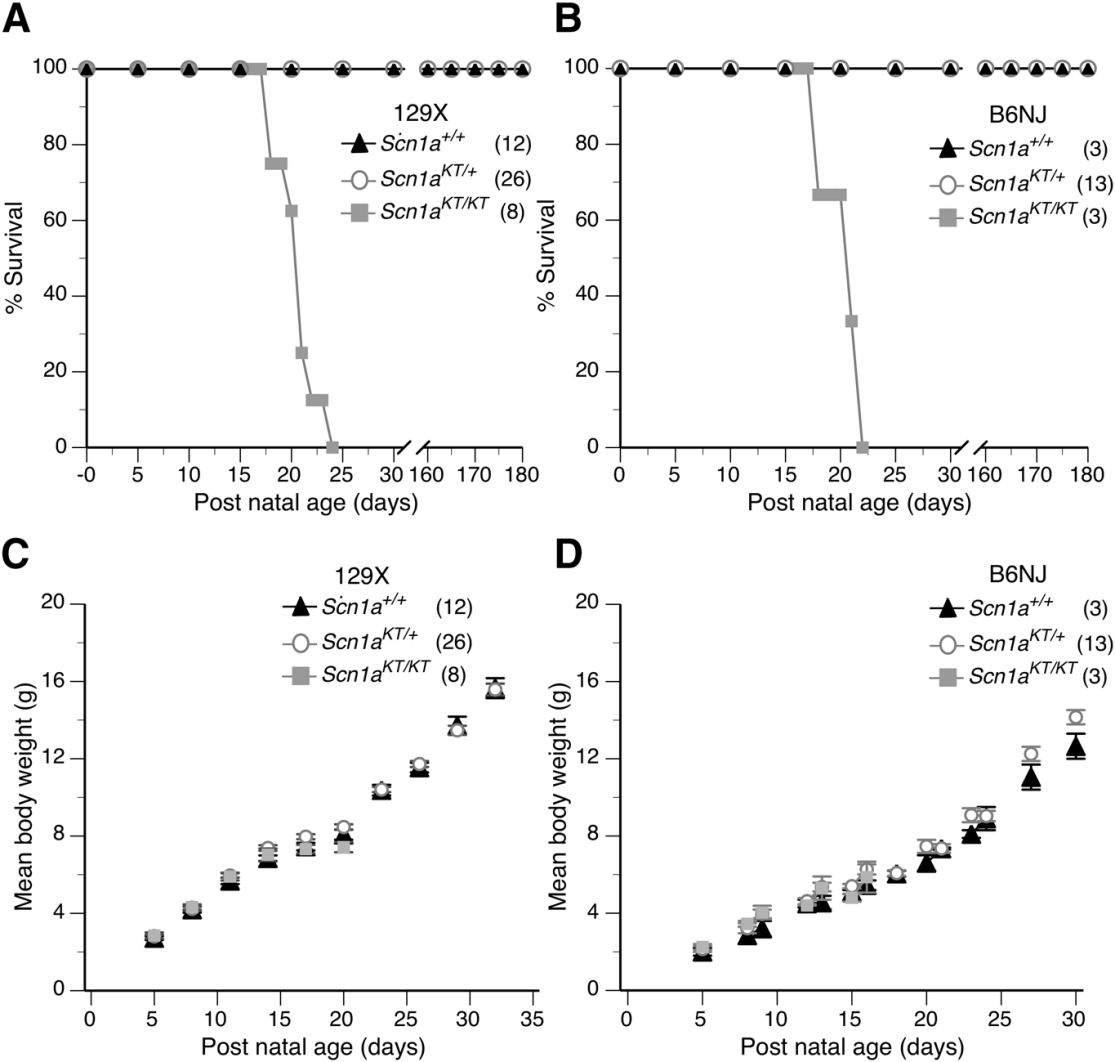
Homozygous (*Scn1a^KT/KT^*) mice have a shortened lifespan in both genetic backgrounds. ***A***, ***B***, Survivorship plots of wild-type (*Scn1a^+/+^*) and mutant mice over a period of one month in 129X1 and B6NJ strains, respectively. Homozygous (*Scn1a^KT/KT^*) mice in both backgrounds displayed reduced mean lifespan (129X1 = 19.9 d; B6NJ = 20.3 d) compared with heterozygous (*Scn1a^KT/+^*) and wild-type (*Scn1a^+/+^*) littermates assayed in parallel. ***C***, ***D***, Body weight of mutant mice (*Scn1a^KT/+^* and *Scn1a^KT/KT^*) was not different from wild-type (*Scn1a^+/+^*) littermates before the age of P20 (Mann–Whitney test, *p *<* *0.05). Data are represented as mean ± SEM. Individual data points of mouse survival and body weights observed across time is listed in detail in Extended Data Table 2-1. *Figure Contributions:* Antara Das performed the experiment and analyzed the results.

Some *SCN1A* mutations that lead to either Dravet syndrome (DS) or GEFS+ epilepsy have been correlated with premature death in mouse models (Yu et al., 2006; Ogiwara et al., 2007; Martin et al., 2010). To determine whether the *Scn1a^KT^* mutation affected longevity, life-span assays were conducted. Homozygous mutants (*Scn1a^KT/KT^*) in both genetic backgrounds died between P17–P25 with average lifespan of 19.6 ± 0.6 and 19.3 ± 1.2 d for 129X1 and B6NJ strains, respectively (Fig. 2*A*,*B*). The lifespan of the homozygous mutants is significantly shorter than heterozygous and wild-type littermates in their respective background (Mantel–Cox test, df 2, 129X1 strain: χ^2^ = 81.8; B6NJ: χ^2^ = 24.9, *p *<* *0.0001) There was no difference in the lifespan of heterozygous (*Scn1a^KT/+^*) and wild-type (*Scn1a^+/+^*) littermates in the same background observed up to six months (Fig. 2*A*,*B*; Extended Data Table 2-1).

Spontaneous seizures were observed in some of the homozygous mice during daily monitoring and weighing of animals over the course of the lifespan assay (Movie 1). To determine whether spontaneous seizure activity is correlated with death in the mutant mice, continuous video monitoring was conducted on three litters of 129X1 mice, between ages P18–P25, which included seven homozygous *Scn1a^KT/KT^* mice and their heterozygous mutant and wild-type littermates. All seven homozygous mice examined exhibited multiple spontaneous seizures with excessive jumping, limb jerking, falling on side, hind leg extension characteristics of classic tonic-clonic seizures (i.e., Racine score 5; Movie 1). All seven homozygous mice died immediately following spontaneous seizure events. No spontaneous seizures were observed in any of the heterozygous mutant or wild-type littermates during the same time period.

Movie 1. Homozygous mouse pup shows spontaneous seizures. 10.1523/ENEURO.0394-20.2021.video.1

Mean body weight of the *Scn1a^KT/KT^*homozygous mice in the 129X1 and B6NJ strains increased at a similar rate to *Scn1a^KT/+^*and *Scn1a^+/+^* littermates between P5 and P16 (multiple *t* tests of two-way ANOVA, *p > *0.05; Fig. 2*C*,*D*). Between P17 and P21, homozygous mutant mice started dying in both the 129X1 and B6NJ strains so it was not possible to do accurate statistical comparisons during this period. There was no difference in the mean body weight of heterozygous *Scn1a^KT/+^*and wild-type *Scn1a^+/+^* animals monitored up to P30–P32 in the 129X1 background (two-way ANOVA, interaction *F*_(9,360)_ = 0.74, *p > *0.05, followed by Sidak’s *post hoc* comparison; Fig. 2*C*) or the B6N background (two-way ANOVA, interaction *F*_(13,161)_ = 0.06, *p > *0.9999, followed by Sidak’s *post hoc* comparison; Fig. 2*D*). Individual data points of mouse body weight across age have been summarized in Extended Data Table 2-1.

### *Scn1a^KT/+^* mice exhibit heat-induced seizures

**Figure 3.**
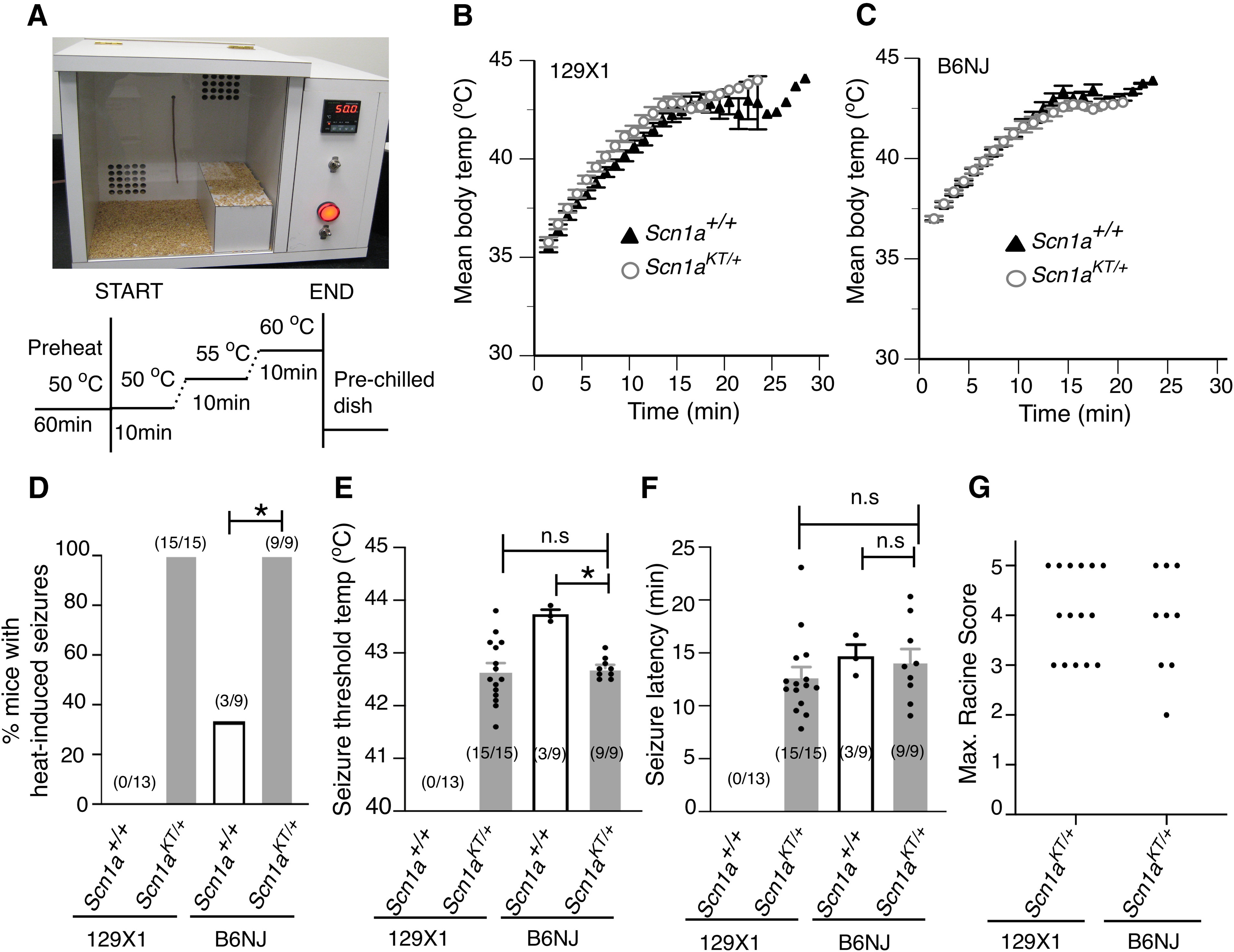
Heterozygous mice (*Scn1a^KT/+^*) exhibit heat-induced seizures and spontaneous seizures. ***A***, Custom-built heating chamber and schematic of the heating protocol used for inducing seizures. ***B***, ***C***, Change in mean body temperature over time in wild-type (*Scn1a^+/+^*) and heterozygous mice (*Scn1a^KT/+^*) in 129X1 and B6NJ strains, respectively. ***D***, Percentage of mice exhibiting heat-induced seizures in both strains. ***E***, Seizure threshold temperature and (***F***) latency to heat-induced seizures in wild-type (*Scn1a^+/+^*) and heterozygous (*Scn1a^KT/+^*) mice in both strains. ***G***, Maximum Racine scores of heat-induced seizures exhibited by heterozygous (*Scn1a^KT/+^*) mice in both genetic strains are shown. Each dot represents maximum Racine score in a single mouse. Data shown in panels ***B–F*** are mean ± SEM. Asterisks (*) indicate significant differences at *p* < 0.05. n.s., not significant. Number of animals are shown in parentheses. Representative electrographic (EEG) traces from a wild-type (*Scn1a^+/+^*) and a heterozygous (*Scn1a^KT/+^*) mouse on 129X1 strain is shown in Extended Data Figure 3-1. *Figure Contributions:* Antara Das and Lisha Zeng performed the heat-induced seizures experiment, Antara Das and An T. Pham performed the EEG experiments, Antara Das analyzed the results.

Pedigree analysis of human families with inherited GEFS+ has shown that individuals heterozygous for *SCN1A* mutations can exhibit a variety of seizure phenotypes with different clinical severity (Abou-Khalil et al., 2001; Zhang et al., 2017). However, febrile seizures are the most common feature of GEFS+ patients, including patients heterozygous for the K1270T mutation. To determine whether the heterozygous mice phenocopy the heat-induced seizures observed in human patients, individual mice (P30–P40) were fitted with a rectal temperature probe before being placed into a custom-built chamber preheated to 50°C (Fig. 3*A*, top panel). By slowly changing the chamber temperature using the heating protocol shown in Figure 3*A*, mouse body temperature was increased at the rate of ∼0.3°C/min to a final temperature of 44°C, similar to previously published thermal seizure induction protocols (Oakley et al., 2013; Warner et al., 2017). There was no difference in the mean body temperature across time between control *Scn1a^+/+^* and heterozygous *Scn1a^KT/+^* mice in both backgrounds (two-tailed unpaired Student’s *t* test, 129X1, *p *=* *0.783; B6NJ, *p = *0.318; Fig. 3*B*,*C*), indicating no overt changes in thermoregulation in *Scn1a^KT/+^* mice.

10.1523/ENEURO.0394-20.2021.tab2-1 Extended Data Table 2-1 Individual data points for (***A***) survivorship curve and (***B***) mean body weight of mice across postnatal age (days) in both strains, 129 and B6N. na, not applicable; d, dead. M1,2,3…, individual mouse under study. Download Table 2-1, DOCX file.

10.1523/ENEURO.0394-20.2021.f3-1 Extended Data Figure 3-1 Representative electrographic traces from a four-month-old wild-type (*Scn1a^+/+^*) and a heterozygous (*Scn1a^KT/+^*) mouse on 129X1 strain. Left, Normal EEG and EMG traces from a wild-type mouse during baseline activity. Right, Example EEG and corresponding EMG recordings during a spontaneous seizure episode (shaded box) in the heterozygous mouse. The seizure episode, highlighted in the shaded box, shows a classic EEG waveform with high amplitude and high frequency polyspike discharges is expanded below. This animal experienced an average of 18.1 ± 5.3 seizures per day with a mean seizure duration of 38.9 ± 6.2 s. Download Figure 3-1, DOCX file.

In the 129X1-enriched strain, no wild-type *Scn1a^+/+^* mouse exhibited heat-induced seizures. In contrast, under identical conditions, all heterozygous *Scn1a^KT/+^* mice had heat-induced seizures (Fig. 3*D*). In the B6NJ background, a small percentage (33%) of the wild-type *Scn1a^+/+^* mice exhibited heat-induced seizures while all heterozygous *Scn1a^KT/+^* mice had heat-induced seizures (Fig. 3*D*; Movie 2). The percentage of animals that had heat-induced seizures was significantly higher in heterozygous mutants compared with wild-type mice in both backgrounds (Fisher’s exact test, 129X1 *p *<* *0.0001; B6NJ *p *=* *0.009). There was no difference in the percentage of animals displaying heat-induced seizures between heterozygous *Scn1a^KT/+^* mice in the 129X1 versus the B6NJ background (Fisher’s exact test, *p *>* *0.999; Fig. 3*D*).

Movie 2. Heterozygous mouse shows heat-induced seizures. 10.1523/ENEURO.0394-20.2021.video.2

Average seizure threshold temperature was similar in the heterozygous *Scn1a^KT/+^* mice in both backgrounds: 129X1, 42.6 ± 0.20°C and B6NJ, 42.7 ± 0.06°C (two-tailed unpaired Student’s *t* test, *p *=* *0.782; Fig. 3*E*). In the small number of B6NJ wild-type mice that did have seizures (three out of nine mice), the mean seizure threshold temperature was significantly higher than the heterozygous mice: *Scn1a^+/+^*, 43.7 ± 0.08°C versus *Scn1a^KT/+^*, 42.7 ± 0.06°C (two-tailed unpaired Student’s *t* test, *p *<* *0.0001; Fig. 3*E*). Seizure latency is defined as the time from introducing animals into the preheated chamber to the onset of the first behavior seizure. There was no difference in the latency to seizure onset between heterozygous mice in either strain: 129X1, 12.7 ± 0.9 min and B6NJ, 14.1 ± 1.2 min (Mann–Whitney test, *p *=* *0.290; Fig. 3*F*). Similarly, latency to seizure onset was not different between wild-type (*Scn1a^+/+^*, 14.7 ± 1.1 min) and heterozygous mice (*Scn1a^KT/+^*, 14.1 ± 1.2 min) on B6NJ background (two-tailed unpaired Student’s *t* test, *p *=* *0.813; Fig. 3*F*).

The seizure severity in each mouse was scored on a modified Racine scale (Cheah et al., 2012). No difference was observed in the maximum Racine score, representative of the most severe heat- induced seizure behavior, between heterozygous *Scn1a^KT/+^* mice in both backgrounds (Mann–Whitney test, *p* > 0.9999; Fig. 3*G*). Mice would typically begin to exhibit heat-induced seizures with head-nodding (Racine score 2) and rapidly progress to forelimb clonus, falling on sides, hindlimb extension and/or uncontrolled jumping (Racine score 3–5). Approximately 40% of heterozygous mice tested on 129X1 (six out of 15) and B6NJ (four out of nine) backgrounds exhibited severe seizure phenotypes (Racine score of 5) characterized by uncontrolled jumping and/or generalized tonic-clonic seizures. Taken together, our data demonstrate that all heterozygous mutant mice exhibit heat-induced seizures with similar frequency, seizure threshold, latency, and seizure severity in a strain-independent manner.

The *Scn1a^KT/+^* mice were also screened for spontaneous seizures via continuous EEG monitoring over a 7-d period. EEG monitoring was performed on adult mice on the 129X1-enriched background between the age of three to four months. One out of five heterozygous (*Scn1a^KT/+^*) animals exhibited spontaneous seizures whereas none of the wild-type littermate (*Scn1a^+/+^*) animals examined in parallel (*n* = 5) displayed spontaneous seizures (Extended Data Fig. 3-1). These results suggest that the *Scn1a^KT^* mutation can lead to a spontaneous seizure phenotype in some heterozygous animals, consistent with some individuals with the K1270T mutations exhibiting afebrile seizures (Abou-Khalil et al., 2001).

### Impaired excitability of PV-positive interneurons in *Scn1a^KT/+^* mice

**Figure 4.**
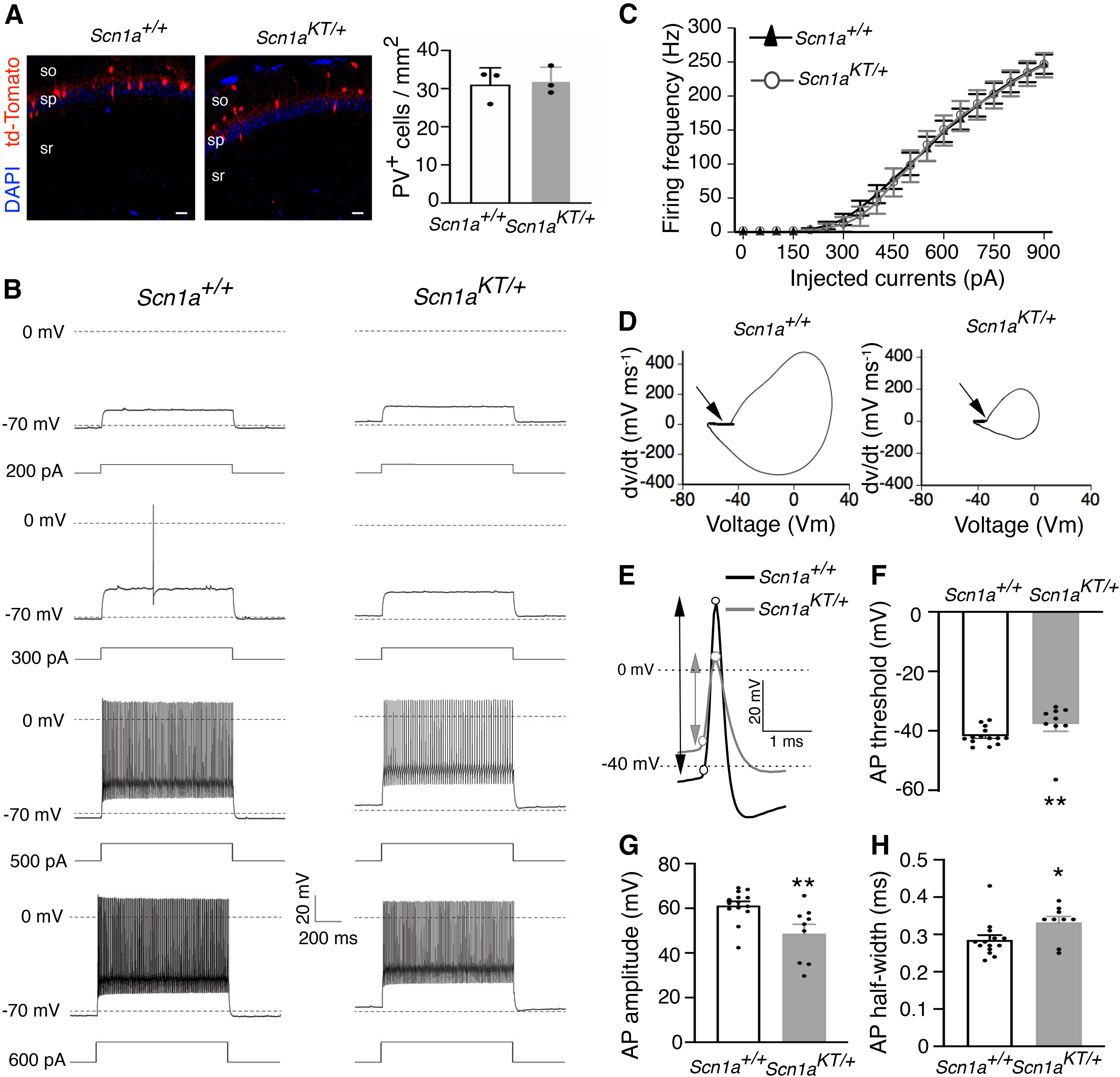
Reduced excitability of PV interneurons in *Scn1a^KT/+^*mice. ***A***, left, Representative wild-type and heterozygous mouse brain sections depicting td-Tomato labeled PV interneurons (red) and DAPI (blue). so, stratum oriens; sp, stratum pyramidale; sr, stratum radiatum. Scale bar: 50 μm. Co-immunolabeling of td-Tomato positive PV interneurons with anti-PV antibody is shown in Extended Data Figure 4-1. ***A***, right, Mean cell density of td-Tomato-labeled PV interneurons is not different between wild-type and heterozygous mice (*n* = 3 mice per genotype, two tailed Student’s *t* test). ***B***, Representative traces of PV interneurons from wild-type (*Scn1a^+/+^*) and heterozygous (*Scn1a^KT/+^*) littermates at different current injection steps. ***C***, Input-output curves showing AP firing frequency in PV interneurons against current injection steps between 0 and 900 pA, data points for 50-pA step increment is shown here. No difference in PV firing frequency between *Scn1a^KT/+^*(gray curve, open circles) and wild-type *Scn1a^+/+^*mice (black curve, triangles) was seen (two-way ANOVA with repeated measures, *p *>* *0.05). ***D***, Phase plots of the first derivative (dv/dt) was plotted against membrane potential (Vm) for the representative AP traces shown in panel ***E***. Arrow indicates the AP threshold. ***E***, Expanded representative traces of first AP fired from a PV interneuron recorded from a *Scn1a^+/+^*and *Scn1a^KT/+^*mouse. Circles represent the AP threshold determined from phase plots in panel ***D*** and the peak of action potentials. Arrows represent AP amplitude. ***F***, AP threshold is more depolarized in *Scn1a^KT/+^* mice compared with wild-type *Scn1a^+/+^* mice. AP amplitude is also reduced in *Scn1a^KT/+^* mice compared with *Scn1a^+/+^* littermates. ***G***, AP half-width is increased in *Scn1a^KT/+^* mice. ***F–H***, All statistical comparisons done by Mann–Whitney test. Data shown are average of 14 and 9 cells from *Scn1a^+/+^* and *Scn1a^KT/+^* littermates, respectively, recorded from at least three different mice. Data are represented as mean ± SEM. Asterisks indicate data means are significantly different. * represents *p* < 0.05, and ** represents *p* < 0.01. *Figure Contributions:* Antara Das did the PV cell counts, Bingyao Zhu performed the electrophysiological recordings, Bingyao Zhu and Antara Das analyzed the results.

Several mutations in *Scn1a* have been associated with reduced excitability of PV-expressing inhibitory interneurons in mouse models of DS (Yu et al., 2006; Ogiwara et al., 2007; Cheah et al., 2012; Tai et al., 2014; Rubinstein et al., 2015) and GEFS+ (R1648H) disorders (Martin et al., 2010; Hedrich et al., 2014). Reduced excitability of inhibitory neurons has also been implicated in a *Drosophila* model of the K1270T mutation (Sun et al., 2012). To examine the effect of K1270T mutation on PV interneurons, *Scn1a^KT/+^*mice were crossed with PV-Cre mice and Ai14-td-Tomato reporter on a C57BL/6J background (i.e., to generate *Scn1a^KT/+^*;PV-Cre;Ai14-td-Tomato mice). Genetic labeling of PV neurons with td-Tomato was confirmed by immunostaining brain slices with anti-PV antibody and nuclei marker DAPI (Extended Data Fig. 4-1). No difference was observed in mean cell density of td-Tomato labeled PV interneuron in CA1 hippocampus between wild-type and heterozygous mice (two-tailed unpaired Student’s *t* test, *p *=* *0.791; Fig. 4*A*).

10.1523/ENEURO.0394-20.2021.f4-1 Extended Data Figure 4-1 A representative PV interneuron (arrow) which was genetically labeled with td-Tomato (magenta) in *Scn1a^KT/+^;PV-Cre;Ai14-tdTomato* mice and co-immunostained with anti-PV antibody (green) and DAPI (blue). Right most panel shows the merged image. Scale bar: 50 μm. Download Figure 4-1, DOCX file.

To evaluate firing properties of the inhibitory PV interneurons, acute slices of the mouse hippocampus were prepared from P30–P40 mice and whole-cell current clamp recordings were obtained from td-Tomato+ neurons from hippocampal CA1. Characteristic fast spiking firing patterns were observed in td-Tomato+ neurons in both *Scn1a^+/+^*and *Scn1a^KT/+^*mice (Fig. 4*B*). Curves of firing frequency versus injected currents in PV interneurons were not significantly different between the *Scn1a^KT/+^*(Fig. 4*C*, gray, open circles) and *Scn1a^+/+^*mice (Fig. 4*C*, black, triangles; two-way ANOVA with repeated measures, interaction: *F*_(90,1890)_ = 0.1346, *p *>* *0.9999). However, a comparison of single action potential properties of PV interneurons revealed differences between *Scn1a^KT/+^*and *Scn1a^+/+^*mice (Table 2; Fig. 4*D–H*). AP threshold was estimated from phase plots of first derivative (dv/dt) of the first ever AP fired (Fig. 4*D*). Although both heterozygous and wild-type PV interneurons fired brief action potentials with a rapid afterhyperpolarization (Fig. 4*E*), the action potential threshold in *Scn1a^KT/+^*was more depolarized than *Scn1a^+/+^*mice (Mann–Whitney test, *p *=* *0.0069; Table 2; Fig. 4*F*). Also, action potential amplitude in *Scn1a^KT/+^*was reduced compared with wild-type *Scn1a^+/+^*(Mann–Whitney test, *p *=* *0.005; Table 2; Fig. 4*E*,*G*). Action potential half-width was broader in *Scn1a^KT/+^*compared *Scn1a^+/+^*mice (Mann–Whitney test, *p *=* *0.031; Table 2; Fig. 4*H*). There was no significant difference in spike frequency adaptation (ISI_1_/ISI_n_) or in the other intrinsic membrane properties measured including whole-cell capacitance, input resistance and rest membrane potential (RMP) between *Scn1a^+/+^*and *Scn1a^KT/+^*mice (Table 2). These results suggest that the K1270T GEFS+ mutation leads to significant impairment in action potential firing of PV interneurons without altering the relationship of firing frequency versus injected currents.

**Table 2:** Electrophysiological properties of PV interneurons recorded from heterozygous (*Scn1a^KT/+^*) and control (*Scn1a^+/+^*) mice, respectively

Properties	*Scn1a^+/+^*	*Scn1a^KT/+^*	*p* value
Cells # (animals #)	14 (5)	9 (3)	
Capacitance (pF)	45.58 ± 9.16	60.0 ± 18.69	0.402
Input resistance (MΩ)	111.79 ± 21.86	74.83 ± 8.94	0.477
Resting potential (mV)	−65.14 ± 0.61	−63.88 ± 0.82	0.227
AP threshold (mV)	−41.79 ± 0.77	−37.70 ± 1.28**	0.0069
AP amplitude (mV)	61.24 ± 1.88	48.26 ± 4.01**	0.005
AP half-width (ms)	0.285 ± 0.01	0.332 ± 0.01*	0.031
Spike Frequency Adaptation (ISI_1_/ISI_n_)	0.820 ± 0.11	0.883 ± 0.19	0.926

Statistical comparisons were made with unpaired Mann–Whitney test except resting membrane potential that was compared with Student’s unpaired *t* test; *p < 0.05 and **p < 0.01.

### Excitability of CA1 pyramidal neurons is unaltered in *Scn1a^KT/+^* mice

**Figure 5.**
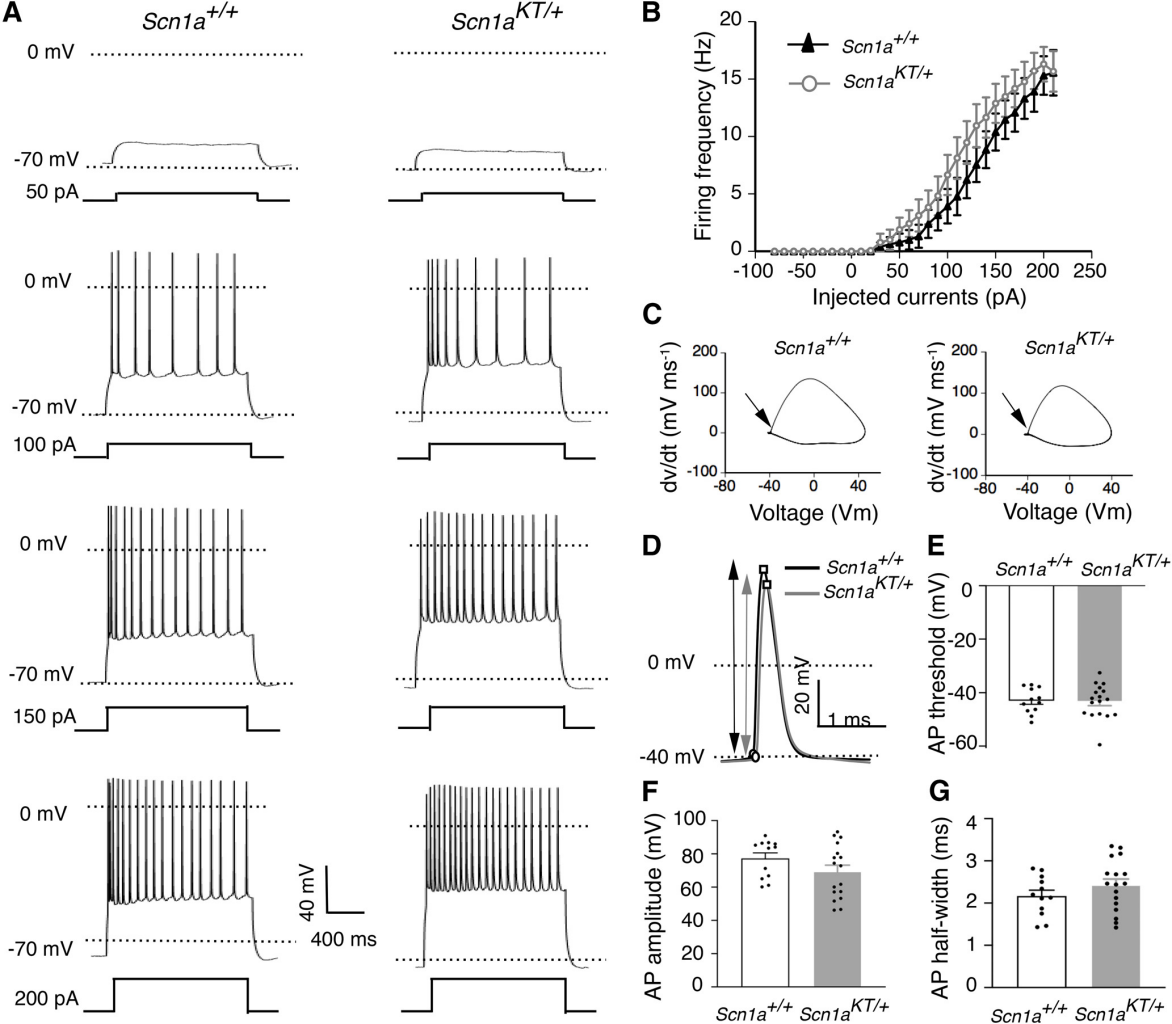
Firing property of CA1 excitatory neurons in *Scn1a^KT/+^* mice remains unaltered. ***A***, Representative traces of action potential firing from CA1 excitatory cells in wild-type (*Scn1a^+/+^*) and heterozygous (*Scn1a^KT/+^*) mice in response to increasing current injection steps. ***B***, No difference in firing frequency at a series of current injection steps was found between heterozygous *Scn1a^KT/+^* mice and *Scn1a^+/+^* littermates (two-way ANOVA, *p *>* *0.05). ***C***, Phase plots (dv/dt vs membrane potential) of the representative AP traces shown in panel ***D***. Arrow indicates the AP threshold. ***D***, Expanded single AP traces from *Scn1a^+/+^* and *Scn1a^KT/+^* mice. Circles represent the inflection point (AP threshold) and the peak of action potentials, arrow indicate AP amplitude. ***E–G***, No change observed in AP threshold, AP amplitude, and AP half-width between heterozygous *Scn1a^KT/+^* and *Scn1a^+/+^* mice (two-tailed unpaired Student’s *t* test, *p *>* *0.05). Data shown is average of 12 and 17 cells from *Scn1a^+/+^* and *Scn1a^KT/+^* littermates, respectively, recorded from at least eight different mice. Data represented as mean ± SEM. *Figure Contributions:* Antara Das and Yunyao Xie performed the experiments, Antara Das analyzed the results.

Although hyperexcitability in *Scn1a* epilepsy mouse models has been largely associated with impaired firing in inhibitory neurons (Yu et al., 2006; Ogiwara et al., 2007; Martin et al., 2010; Cheah et al., 2012; Dutton et al., 2013; Hedrich et al., 2014; Tai et al., 2014; Rubinstein et al., 2015), several studies suggest that excitatory neuronal firing could also be altered as seen in a human iPSC *SCN1A* model (Liu et al., 2013) and an *SCN2A* mouse model (Ogiwara et al., 2018). Action potential firing was recorded in excitatory hippocampal CA1 pyramidal neurons from adult (P28–P40) mouse brain slices. Representative traces from wild-type *Scn1a^+/+^*and heterozygous *Scn1a^KT/+^* mice show regular spiking firing pattern with characteristic AP-doublet as seen in the 100-pA step (Fig. 5*A*). Firing frequency versus injected current curves are not significantly different between *Scn1a^KT/+^*(Fig. 5*B*, gray curve, open circles) and the *Scn1a^+/+^*littermates (Fig. 5*B*, black curve, triangles; two-way ANOVA with repeated measures, interaction: *F*_(28,756)_ = 0.6038, *p *=* *0.948). AP threshold values were estimated from phase plots of the first derivative (dv/dt) of AP waveform and were not different between *Scn1a^KT/+^*and *Scn1a^+/+^*mice (Fig. 5*C*,*E*). Similarly, there was no significant difference in AP amplitude or AP half-width, or spike frequency adaptation (ISI_1_/ISI_n_) between *Scn1a^KT/+^*and *Scn1a^+/+^*mice (two-tailed unpaired Student’s *t* test, *p *>* *0.05; Fig. 5*F*,*G*; Table 3). There was no difference in other membrane properties including cell capacitance, input resistance, and RMP (two-tailed unpaired Student’s *t* test, *p *>* *0.05; Table 3). These results suggest that the K1270T mutation, similar to other *Scn1a* mouse models, preferentially impairs firing of inhibitory interneurons in hippocampal CA1 (Yu et al., 2006; Ogiwara et al., 2007; Martin et al., 2010; Cheah et al., 2012; Dutton et al., 2013; Tai et al., 2014; Hedrich et al., 2014; Rubinstein et al., 2015).

**Table 3:** Electrophysiological properties of excitatory CA1 pyramidal neurons from heterozygous (*Scn1a^KT/+^*) and control (*Scn1a^+/+^*) mice

Properties	*Scn1a^+/+^*	*Scn1a^KT/+^*	*p* value
Cells # (animals #)	12 (8)	17 (9)	
Capacitance (pF)	27.71 ± 1.93	31.77 ± 3.23	0.340
Input resistance (MΩ)	182.04 ± 20.59	187.62 ± 13.58	0.815
Resting potential (mV)	−65.16 ± 1.01	−65.09 ± 0.69	0.947
AP threshold (mV)	−42.99 ± 1.36	−43.29 ± 1.54	0.891
AP amplitude (mV)	77.27 ± 3.30	69.28 ± 3.90	0.152
Spike frequency adaptation (ISI_1_/ISI_n_)	0.234 ± 0.04	0.384 ± 0.07	0.085

Statistical comparisons were made with two-tailed unpaired Student’s *t* test

## Discussion

### Seizure behavior

To our knowledge, this is the first study reporting the use of CRISPR/Cas9-based gene-editing to generate a mouse model of human GEFS+ epilepsy. All mice heterozygous for the K1270T GEFS+ mutation, in both the 129X1 and the B6NJ background, exhibited heat-induced seizures. This is consistent with the febrile seizure phenotype observed in the majority of patients with one copy of the K1270T mutant allele (Abou-Khalil et al., 2001). The seizure threshold temperature of heterozygous *Scn1a^KT/+^* mice (42–43°C) is higher than the typical body temperature of human patients during fever. However, this is not unexpected given a previous report demonstrating heat-induced seizure behavior at similarly elevated temperatures in another mouse GEFS+ model, R1648H *Scn1a^RH/+^* (Martin et al., 2010). While there is potential for off-target mutations associated with CRISPR editing, it seems unlikely this contributes to the seizure phenotype based on DNA sequencing indicating there was no evidence of mutant sequences at the three most likely off-target sites. These findings, together with the observation that seizures occurred at significantly lower temperature threshold in *Scn1a^KT/+^* mice compared with their wild-type littermates, strongly supports the hypothesis that the presence of one copy of the K1270T mutation results in the heat-induced seizure phenotype.

We also report spontaneous seizure behavior, based on continuous EEG recordings, in one of five of the heterozygous *Scn1a^KT/+^* mutants, that was not observed in five wild-type littermates monitored in parallel. While the sample size is small, this finding is similar to results in the R1648H GEFS+ mouse model where two of 14 *Scn1a^RH/+^* mice exhibited spontaneous seizures while none was observed in wild-type littermates (Martin et al., 2010). The result in the *Scn1a^KT/+^* genotype is not unexpected since some, but not all, of the patients with the K1270T GEFS+ patients exhibit spontaneous as well as febrile seizures (Abou-Khalil et al., 2001). Analysis on larger sample size of animals will be required to describe the range of spontaneous seizure behaviors associated with the K1270T mutation.

Unlike the human population where all individuals with the K1270T mutation are heterozygous, the mouse model also provides the opportunity to evaluate animals homozygous for the mutant gene. Consistent with other mouse models of GEFS+ or DS, homozygous *Scn1a^KT/KT^*mice display a dramatically reduced life span compared with the heterozygous mutant and wild-type littermates (Yu et al., 2006; Ogiwara et al., 2007; Martin et al., 2010; Cheah et al., 2012). In contrast to the study of DS mice (Yu et al., 2006) there was no difference in lifespan between the mutant mice on C57BL/6NJ and 129X1/SvJ backgrounds. However, similar to the other models, continuous video monitoring revealed recurrent spontaneous seizures in the *Scn1a^KT/KT^* mice just before death (Yu et al., 2006; Ogiwara et al., 2007; Cheah et al., 2012). Sudden unexpected death in epilepsy (SUDEP) is known to occur, possibly because of lethal cardiac arrhythmias following severe seizures in *Scn1a* knock-in R1407X DS mouse model (Auerbach et al., 2013) and *Scn1a*+/− knock-out DS mouse model (Kalume et al., 2013). Future studies that include simultaneous EEG and electrocardiogram monitoring could help determine whether cardiac dysfunction is associated with death following spontaneous seizures in the *Scn1a^KT/KT^*mice. It will also be interesting to determine whether heat-induced seizures in juvenile homozygous mutant mice (evaluated before their death) have reduced seizure threshold and seizure latency compared with the heterozygous mutants as seen in R1648H GEFS+ mouse model (Martin et al., 2010).

In addition to heat-induced/spontaneous seizures and reduced lifespan in homozygous mutants, the R1648H mutation in knock-in mice also results in mild impairment in social behavior and learning/memory tasks (Sawyer et al., 2016). Future studies on the K1270T knock-in mice will include evaluation of sleep/wake cycles, learning/memory and cognitive behavior, social interaction, and effects of stressors. Comparison with R1648H GEFS+ and other existing mouse models of epilepsy will be informative in identifying both common and distinct features associated with specific mutations.

### Alterations in neuronal firing in *Scn1a^KT/+^* mice

Reduced excitability of PV interneurons has been implicated in contributing to hyperexcitable circuits in *Scn1a* mouse models of DS and GEFS+ (Dutton et al., 2013; Hedrich et al., 2014; Tai et al., 2014; Rubinstein et al., 2015; Favero et al., 2018). The selective impairment of evoked firing in hippocampal CA1 PV-expressing inhibitory neurons in the *Scn1a^KT/+^* mice is consistent with these previous models. However, the mouse hippocampus also contains a variety of inhibitory GABAergic interneuron subtypes including somatostatin (SST) and vasoactive intestinal peptide (VIP)-expressing neurons, that play an important role in microcircuit activity (Pelkey et al., 2017; Soltesz and Losonczy, 2018). Dysfunction in one or more of these inhibitory neuron populations could also disrupt both the feedback and feedforward inhibitory circuitry, contributing to seizure activity (Paz and Huguenard, 2015; Khoshkhoo et al., 2017). NaV1.1 is predominantly localized in the soma and in the axon initiating segment of PV-positive neurons (Ogiwara et al., 2007) but it is also present in SST and VIP neurons (Ogiwara et al., 2007; Li et al., 2014; Tai et al., 2014; Goff and Goldberg, 2019). One study showed that selective deletion of Nav1.1 in PV or SST-expressing neurons resulted in reduced neuronal excitability but led to phenotypically distinct behavioral disorders, suggesting distinct roles of interneurons subpopulations in mediating DS phenotypes (Rubinstein et al., 2015). In addition, irregular spiking of VIP interneurons has been associated with DS phenotypes in an *Scn1a*+/− mouse model (Goff and Goldberg, 2019). These studies emphasize the need for future work focused on multiple subtypes of interneuron populations to better understand how the K1270T mutation contributes to seizure phenotypes in *Scn1a* mouse models.

The excitability of GABAergic PV interneurons was impaired in heterozygous *Scn1a^KT/+^*mice as reflected in reduced AP amplitude and depolarized AP threshold. This is similar to the finding of APs with reduced amplitude and broadened half-width in hippocampal interneurons of heterozygous and homozygous *Scn1a* knock-out mice (Yu et al., 2006). However, unlike the inhibitory neurons in the R1648H GEFS+ mouse model, where neuronal firing frequency was reduced in the mutants (Martin et al., 2010), the I/F relationship in PV interneurons remained unchanged in K1270T heterozygous mutant mice despite the changes in AP properties. A previous study of hippocampal bipolar neurons in a rat model of *SCN1A* N1417H GEFS+ disorder also reported a reduction in AP amplitude without alteration in AP frequency (Mashimo et al., 2010). In these cases, it is possible that the voltage dependence of activation and steady-state inactivation are altered in opposite directions and thus, “cancel out” their effects on the I/F relationship. It is also possible the unchanged I/F relationship is because of compensatory alterations in the number and/or locations of ion channels encoded by other genes.

The present study did not evaluate the properties of the underlying sodium currents. However, studies in heterologous systems have shown that missense mutations located in transmembrane segments can lead to misfolding of the sodium channel assembly (Rusconi et al., 2007, 2009; Bechi et al., 2015; for review, see Terragni et al., 2018; Mantegazza and Broccoli, 2019). The *Scn1a* K1270T missense mutation, located in the S2 transmembrane segment of domain DIII, could lead to misfolding of the Nav1.1 channel in a way that alters voltage-dependent gating properties and/or trafficking of the channel, which in turn could contribute to the reduced AP amplitude. In the future, analysis of the sodium current properties and high resolution cryo-EM or x-ray diffraction data will be important in determining how the subcellular changes that contribute to reduced excitability.

Since the K1270T mouse model displays heat-induced seizures at high body temperatures, future studies to determine how elevated temperatures affect the firing properties and sodium channel function in PV neurons will be important to further understand the seizure mechanism. In addition, a detailed study of synaptic transmission in the hippocampal circuit at permissive and nonpermissive temperature will be critical in understanding the effects of K1270T mutation on the E/I balance in the brain.

### Comparison of cellular mechanisms in three K1270T knock-in model systems

Elucidating the cellular machinery that regulates neuronal function in taxonomically diverse models can help identify both conserved and distinct pathways that lead to seizure phenotypes, an important step toward developing patient-specific therapies. A previous study has generated a knock-in *Drosophila* line carrying the same K1270T mutation in *para*, the only sodium channel gene in flies. In addition, isogenic pairs of hiPSC-derived neurons carrying the K1270T mutation have also been developed. Common alterations in all three models provide insight into how the *SCN1A* K1270T mutation gives rise to seizures. Comparing the findings of the iPSC, fly and mouse models consistently reveal that the K1270T mutation leads to impaired evoked firing properties of inhibitory neurons (Sun et al., 2012; Xie et al., 2020). This mutation also results in reduced spontaneous firing in inhibitory neurons in the fly (Sun et al., 2012). Reduced inhibition may result in an imbalance of excitation and inhibition in the neural network, as shown in the iPSC model, which may further induce seizure generation as shown in the fly and mouse models.

However, the specific alterations in sodium currents associated with impaired firing in inhibitory neurons appear to be different in the three models, identifying distinct cellular mechanisms that lead to heat-induced seizure phenotypes. In the K1270T mutant fly model, reduced firing in inhibitory neurons at elevated temperature is associated with a temperature-dependent hyperpolarized shift in the deactivation threshold of persistent sodium currents (Sun et al., 2012). Reduced firing frequency and AP amplitude in iPSC-derived inhibitory neurons carrying the K1270T mutation, even in the absence of elevated temperature, is associated with a constitutive reduction in sodium current density (Xie et al., 2020). In the current study of the K1270T mouse model, PV inhibitory firing frequency is not reduced in recordings made at room temperature. However, excitability is impaired based on a depolarizing shift in the AP threshold and a reduction in the AP amplitude. This suggests that the K1270T mutation in mice, like human iPSC-derived neurons, might result in constitutive reduction in sodium currents that makes PV interneurons less excitable.

Future studies that examine sodium current properties in K1270T mutant mice, particularly focused on the effects of elevated temperature, may reveal additional alterations in persistent sodium current properties observed in the *Drosophila* model. A recent study of the DS mutation H939R in the *SCN1A* gene in a mouse model and patient hiPSC-derived mixed forebrain neurons, found dysfunction of Nav1.1 channels lead to alterations in the electrophysiological properties of excitatory and inhibitory neurons in both models (Dyment et al., 2020). The mutant mice replicated typical features of DS phenotypes including reduced longevity, susceptibility to spontaneous and heat-induced seizures, but also included the novel finding of reduced acetylcholine expression in hippocampal neurons (Dyment et al., 2020). This demonstrates the importance of studying novel mouse models to identify distinct cellular mechanisms that contribute to mutation-specific disease phenotypes.

The K1270T mutant mouse model generated for the present study provides insights into how this mutation alters activity in behaviourally relevant circuits which is not possible in the *Drosophila* or hiPSC GEFS+ models. Experimental animal models and clinical studies, have demonstrated that enhancing serotonin (5-HT) levels in the brain inhibits hyperexcitability, suggesting that 5-HT receptors can act as potential anti-epileptic drug targets (Igelström, 2012; Schutte et al., 2014; Venzi et al., 2016; Griffin et al., 2018). Both 5-HT receptor agonists or reuptake blockers (such as fluoxetine and citalopram) can have anti-seizure effects (Favale et al., 1995, 2003; Albano et al., 2006; Silenieks et al., 2019). A previous study has shown that enhancing 5-HT levels can suppress sensitivity to heat-induced seizures in a dose-dependent manner in *Scn1a* (S1231R) DS flies but exacerbates heat-induced seizures in K1270T GEFS+ flies (Schutte et al., 2014). Possible mechanism of 5-HT-dependent suppression of seizures involves enhanced spontaneous firing of GABAergic local neuron (LN) that differentially affect seizure susceptibility in DS versus GEFS+ flies (Schutte et al., 2014). It would be interesting to determine whether enhancing 5-HT levels in K1270T mutant mice worsens the heat-induced seizure phenotype (similar to *Drosophila* K1270T model), by performing simultaneous EEG recordings on mice undergoing heat-induced seizures. We speculate that key effects of the mutation that are preserved across multiple species (fruit-flies, mice and humans) are likely to reflect cellular mechanisms contributing to the disease phenotype in human. The *Scn1a* K1270T mouse is the second GEFS+ mouse model to be reported and it closely mimics the human patient phenotype. This new mouse model can further advance our understanding of the variable seizure phenotypes and complex cellular circuitry associated with sodium ion channel mutation-dependent epilepsy.

## Synthesis

Reviewing Editor: Katalin Toth, University of Ottawa Decisions are customarily a result of the Reviewing Editor and the peer reviewers coming together and discussing their recommendations until a consensus is reached. When revisions are invited, a fact-based synthesis statement explaining their decision and outlining what is needed to prepare a revision will be listed below. The following reviewer(s) agreed to reveal their identity: Viji Santhakumar.

The study describes a new mouse model of sodium channel mutation implicated in epilepsies. This can be a useful tool for future studies. The experiments are generally well done, but reviewers offered several constructive points, addressing these could significantly improve the manuscript. The authors should carefully address these issues identified in the reviewers' reports below.

This is a straightforward report of the generation and initial characterization of a mouse model of GEFs+. The authors introduce the K1270T mutation into the Scn1a gene of the mouse genome using CRISPR technology. Multiple mutations in SCN1a are associated with childhood epilepsies such as Dravet and GEFs and there are already several mouse models available to model these. However, this particular mutation has not been engineered into a mouse previously and this new model may be an additional resource to the field. The authors perform a relatively cursory assessment of the mouse showing that mice homozygous for the mutation have elevated mortality. Het mice on two inbred backgrounds were tested in a febrile seizure model and in both 129 and B6 backgrounds demonstrated increased heat induced seizures. Finally the authors crossed mice with PV-Cre;Ai14 mice to label PV interneurons and recorded their intrinsic properties juvenile mice. AP threshold was more depolarized, amplitude was decreased and half width increased in PV interneurons but not pyramidal neurons. These results are in line with what is already known about Scn1a mutations which affect interneurons specifically because of the normal enrichment of Nav1.1. While there are no major concerns with the data, the scope of the paper is very narrow, and the paper does not go beyond what is already known about Scn1a mouse models.

Beyond that criticism I have only minor points that would require some attention

Rev. #1.

1. The authors should consider including some further discussion of how the mutation in the channel affects the functional/assembly/trafficking properties of the channel.

2. Figure 2 should be removed. It is difficult to see what this adds to the paper.

3. Fig 3: It is difficult to see the symbols for the het mice in these graphs. Please modify.

4. Spontaneous seizures: The authors should consider increasing the cohort or take this analysis out. If the authors decide to keep this information some additional quantification of the EEG would be appropriate.

5. Figure 5 and 6. The authors should include dv/dt phase plots to illustrate Vthr, Vmax

6. Ideally a fuller analysis of synaptic transmission in the slice is required to assess the effect of the altered AP properties in PV interneurons.

7. Figure 6 The authors should measure adaptation of APs in their recordings. At least in the representative examples it looks like there are differences in KT mice

Rev. #2.

This is a solid study in which the authors develop a murine model of a sodium channel mutation (SCN1A K>T) implicated in epilepsies. The mutation was tested in 2 genetic backgrounds for phenotypic changes as well as effects of the mutation on cellular physiology. While mice homozygous for the mutation developed epilepsy and died prior to PD 30, the authors performed long-term analysis of the heterozygous mice and identified temperature induced seizures. Physiological analysis identified a selective increase in action potential threshold in parvalbumin interneurons and not in CA1 pyramidal cells suggesting that decrease in interneuron excitability may underlie the phenotype. This is consistent with findings in other GEFS+/SCN1A mutations. Careful controls including use of two background strains, screening for off-target mutations, videoEEG analysis of phenotype and testing for genotype specific changes in parvalbumin expression are particular strengths of the manuscript. This well validated new mouse model would be an useful tool for the research community.

Specific Comments:

1. The physiological assays were conducted at room temperature, a permissive temperature, and not at physiological temperature when the temperature dependent phenotype may be more obvious.

2. It is surprising that the I/F relationship in PV neurons remains unchanged despite a change in threshold. Is there a change in rheobase? How was AP threshold measured. This is not detailed in the methods? Was there a threshold dV/dt change used to determine AP threshold?

## Author Response

Response to Reviewers

Please note that the page and line numbers mentioned below correspond to the manuscript document

with revised text in colored font.

Reviewer #1

1. The authors should consider including some further discussion of how the mutation in the

channel affects the functional/assembly/trafficking properties of the channel.

Response: We have previously hypothesized that impaired firing seen in PV interneurons in

Scn1a K1270T heterozygous mice arises due to reduction in sodium current based on studies in

Drosophila and a human iPSC model of the K1270T mutation (Sun et al., 2012; Xie et al., 2020).

We have expanded the discussion section on page 25, lines 13-23 to include the possible

outcomes of K1270T missense mutation on sodium channel function, assembly and trafficking.

2. Figure 2 should be removed. It is difficult to see what this adds to the paper.

Response: Modification of an organism’s genome with CRISPR/CAS9 can involve risk of offtarget events. In many cases secondary "off-target" CRISPR sites are located on different

chromosomes which can be removed via backcrossing with a parental wild-type strain animal.

However, due to sodium channels being encoded by a multi-gene family and because Scn3a and

Scn7a are located relatively close to Scn1a on the same chromosome, we felt it prudent to

determine if either gene had also been modified while modifying Scn1a. Indeed, reviewer #2

recognized that our finding that the sequences of the nearby sodium channel genes were not

altered was a strength of the study. However, as this data is not a major focus of the study we

moved old Figure 2 to the extended data where it is presented as new Extended Data Table 1-1

and new Extended Data Figure 1-1. To enable readers to follow the text in the results section on

off-target analysis, we have summarized the results of the off-target analysis in new Table 1 in

the main manuscript.

3. Fig 3: It is difficult to see the symbols for the het mice in these graphs. Please modify.

Response: We have modified the symbols for heterozygous mice. For clarity, the symbols are

now plotted for every 5 days instead of every 1 day, matching the ticks on x-axis representing age

of mice (in days).

4. Spontaneous seizures: The authors should consider increasing the cohort or take this analysis

out. If the authors decide to keep this information some additional quantification of the EEG would

be appropriate.

Response: We believe it is important to retain the EEG data because it indicates that some of the

GEFS+ KT mice (20%) have spontaneous seizures and is consistent with the observation of

spontaneous seizures in some but not all GEFS+ patients carrying this mutation. Further, it is

consistent with the finding of spontaneous seizures in a low percentage of heterozygous mutant

animals in the GEFS+ (R1648H) mouse model. However, we also agree that the sample size is

small (1/5) in our study and therefore we have removed the EEG figure panel from the main text

and it is now included in Extended Data Figure 3-1. We had mentioned this in the discussion on

page 22 lines 12-15 "While the sample size is small, this finding is similar to results in the R1648H

GEFS+ mouse model where 2 of 14 Scn1aRH/+ mice exhibited spontaneous seizures while none

was observed in wild-type littermates (Martin et al., 2010)."

2

5. Figure 5 and 6. The authors should include dv/dt phase plots to illustrate Vthr, Vmax.

Response: We appreciate this suggestion and have re-analyzed AP threshold and AP amplitude

by plotting phase plots of dv/dt vs V. AP threshold was estimated from dv/dt phase plots where

the slope crossed 10 mV/ms value, as previously described in (Favero et al., 2018). Using this

value as AP threshold, AP amplitude was estimated as the difference of the threshold to maximum

peak in first AP fired (described in detail in methods on pages 11-12). Corresponding phase plots

for the representative AP traces have now been added in corresponding figures (now Figure 4

and Figure 5 respectively) to illustrate this.

There was no change in statistical trends or conclusions regarding firing properties of

excitatory or inhibitory neurons, when AP characteristics were compared using method 1 (old) vs

method 2 (phase plots). Relevant sections of main text, figures and tables have been updated

with the new methodology and data.

6. Ideally a fuller analysis of synaptic transmission in the slice is required to assess the effect of

the altered AP properties in PV interneurons.

Response: We agree that detailed analysis of synaptic transmission in mouse brain slices would

add value to our study and we intend to do this in future studies. We have now added text in the

discussion on page 26 lines 4-7 to emphasize that understanding the mechanisms of synaptic

transmission in K1270T heterozygous mice will be important in assessing the effect of the altered

AP firing in PV interneurons.

7. Figure 6 The authors should measure adaptation of APs in their recordings. At least in the

representative examples it looks like there are differences in KT mice

Response: We have now included data on spike frequency adaption (SFA) in CA1 excitatory

neurons. There is no difference between WT and Het mice. SFA was compared by estimating

ratio of inter-spike intervals between the first two and last two spikes (ISI1/ISIn) as previously

described in Favero et al., 2018. We have included this data in the text and Table 3. Additionally,

we find no difference in SFA in PV neurons and this data has been included in Table 2.

Reviewer #2

1. The physiological assays were conducted at room temperature, a permissive temperature,

and not at physiological temperature when the temperature dependent phenotype may be more

obvious.

Response: We agree that electrophysiological recordings at higher temperature will advance our

understanding of cellular mechanisms of K1270T seizures. In our hands, it has been challenging

to stably record neuronal firing at bath temperatures around 35-40 oC but this is one focus of our

future studies. We elaborate on this in the discussion on page 26 lines 1-7.

2. It is surprising that the I/F relationship in PV neurons remains unchanged despite a change in

threshold. Is there a change in rheobase? How was AP threshold measured. This is not detailed

in the methods? Was there a threshold dV/dt change used to determine AP threshold?

3

Response: The I/F relationship in PV interneurons remains unchanged despite a more

depolarized AP threshold in the heterozygous neurons. It is possible that the voltage dependence

of activation and steady-state inactivation are altered in opposite directions and as a result, this

could "cancel out" their effects on the I/F relationship. Therefore, detailed study of the sodium ion

channel function in PV interneurons would be helpful in understanding the effect of Scn1a K1270T

mutation on functional properties of PV interneurons. We have added this to the discussion on

page 25 lines 3-12.

In the previous version of the manuscript, AP threshold was calculated manually as inflection

point on first current step that evokes an AP using Clampfit. As suggested, we re-analyzed AP

threshold and AP amplitude by plotting phase plots of dv/dt vs V. AP threshold was estimated

from dv/dt phase plots where the slope crossed 10 mV/ms value, as previously described in

Favero et al., 2018. Using this value as AP threshold, AP amplitude was estimated as the

difference of the threshold to maximum peak in first AP fired in Clampfit (described in detail in

methods on pages 11-12). Corresponding phase plots for the representative AP traces have now

been added in corresponding figures (now Figure 4 and Figure 5 respectively) to illustrate this.

There was no change in statistical trends or conclusions regarding firing properties of excitatory

or inhibitory neurons, when AP characteristics were compared using method 1 (old) vs method 2

(phase plots). Relevant sections of main text, figures and tables have been updated with the new

methodology and data.

The rheobase, was estimated as the minimum current injected to elicit at the first AP and it was

not different between heterozygous and wild-type mice. The average input resistance is ∼100

Mohm and the difference of AP threshold between two genotypes is ∼4mV. The difference of

rheobase should be ∼40pA and we should be able to see it with our 10pA current steps. One

possible explanation of why we saw no difference could be the large variation in the input

resistance. However, a more accurate way to determine the rheobase is a ramp test which we

did not do, so we have decided to not include the rheobase measurements in the manuscript.

References:

Favero, M., Sotuyo, N.P., Lopez, E., Kearney, J.A., and Goldberg, E.M. (2018). A Transient

Developmental Window of Fast-Spiking Interneuron Dysfunction in a Mouse Model of Dravet

Syndrome. J. Neurosci. Off. J. Soc. Neurosci. 38, 7912-7927.

Martin, M.S., Dutt, K., Papale, L.A., Dubé, C.M., Dutton, S.B., de Haan, G., Shankar, A., Tufik, S.,

Meisler, M.H., Baram, T.Z., et al. (2010). Altered function of the SCN1A voltage-gated sodium

channel leads to gamma-aminobutyric acid-ergic (GABAergic) interneuron abnormalities. J. Biol.

Chem. 285, 9823-9834.

Sun, L., Gilligan, J., Staber, C., Schutte, R.J., Nguyen, V., O’Dowd, D.K., and Reenan, R. (2012).

A knock-in model of human epilepsy in Drosophila reveals a novel cellular mechanism associated

with heat-induced seizure. J. Neurosci. Off. J. Soc. Neurosci. 32, 14145-14155.

Xie, Y., Ng, N.N., Safrina, O.S., Ramos, C.M., Ess, K.C., Schwartz, P.H., Smith, M.A., and

O’Dowd, D.K. (2020). Comparisons of dual isogenic human iPSC pairs identify functional

alterations directly caused by an epilepsy associated SCN1A mutation. Neurobiol. Dis. 134,

104627.
