# Supplementary material for: Interneuron Dysfunction in a New Mouse Model of SCN1A GEFS+
Source: eNeuro. 2021 Apr 8;8(2):ENEURO.0394-20.2021. doi: 10.1523/ENEURO.0394-20.2021 (PMC8174035; doi:10.1523/ENEURO.0394-20.2021)
Supplement: Extended Data Table 1-1 — Primers for PCR amplification and sequencing for off-target analysis. The table lists the genes, exon positions, and sequences of the forward and reverse primers used to screen off-target effects in Scn1a K1270T mice. Fwd, forward; Rev, reverse. Download Table 1-1, DOCX file. [file enu-eN-NWR-0394-20-s02.docx]

| Gene | Exon | Direction | Primer Sequence |
| --- | --- | --- | --- |
| *Scn3a*  ENSMUSG00000057182 | *Scn3a*-001 Exon 20 | Fwd | 5' CTTTGGTCTTTAGCTTCTGCCTGAT 3' |
|  |  | Rev | 5' CTAGGAGTTCCTGTGGACTTTACTG 3' |
| *Scn7a*  ENSMUSG00000034810 | *Scn7a*-001 Exon 18 | Fwd | 5' ACACGCAACCTGCTATTACCTATAC 3' |
|  |  | Rev | 5' TGGGGAGGTCTGGTAGGATGTTAGA 3' |
| *Cd44*  ENSMUSG00000005087 | *Cd44-*001 within intron 3’ | Fwd | 5' TCATTTGAGTGGACACGAGCATGAA3' |
|  |  | Rev | 5' ACACATACAGGCCAGGTCATAGAAG 3' |

Extended Data Table 1-1. Primers for PCR amplification and sequencing for off-target analysis. The table lists the genes, exon positions and sequences of the forward and reverse primers used to screen off-target effects in *Scn1a* K1270T mice. Fwd and Rev are abbreviated for forward and reverse set of primers.
