# Supplementary figures and images for: Interneuron Dysfunction in a New Mouse Model of SCN1A GEFS+
Source: eNeuro. 2021 Apr 8;8(2):ENEURO.0394-20.2021. doi: 10.1523/ENEURO.0394-20.2021 (PMC8174035; doi:10.1523/ENEURO.0394-20.2021)

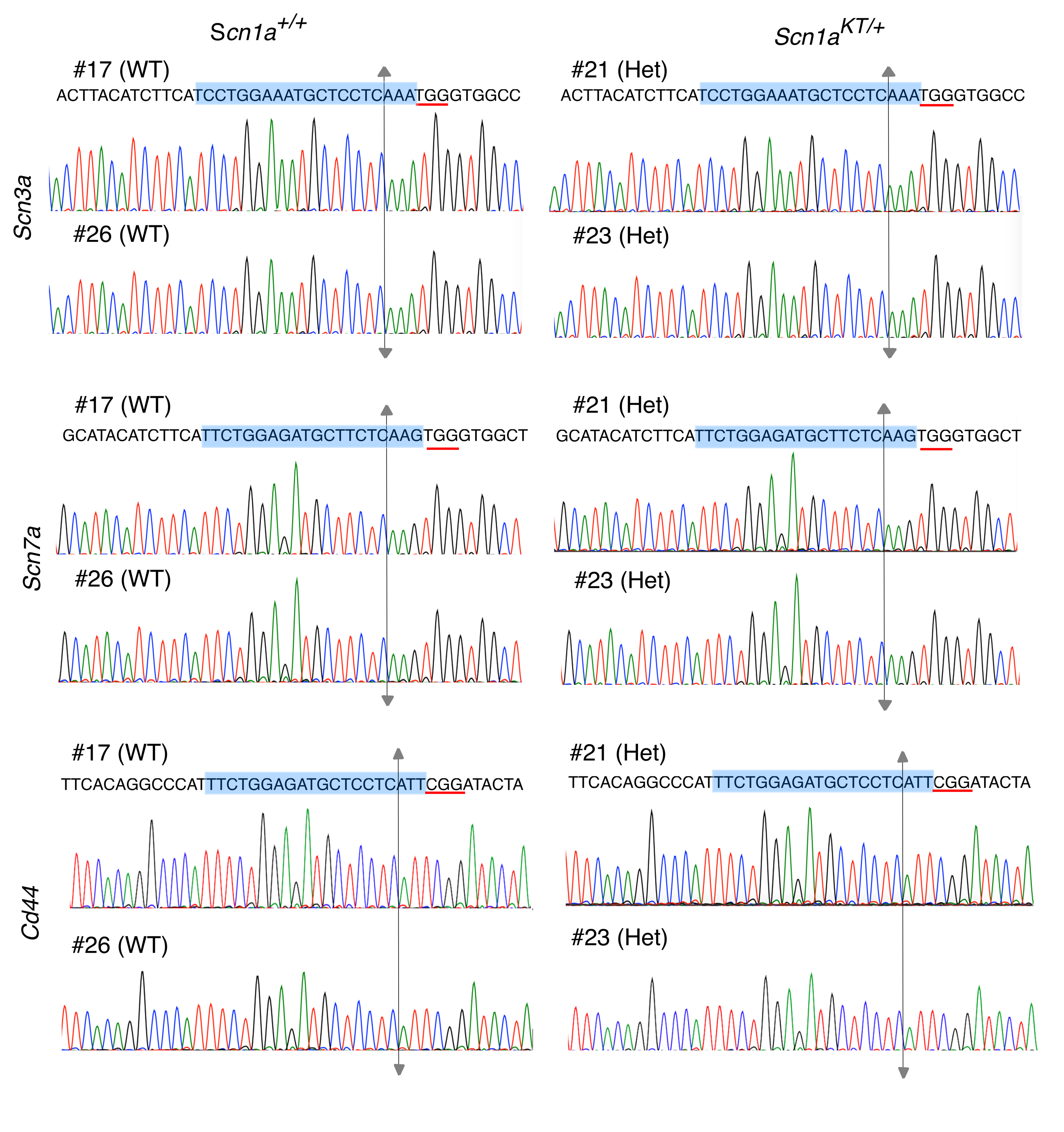


Extended Data Figure 1-1

Supplement: Extended Data Figure 1-1 — Analysis of off-target effects. Chromatograms of DNA sequence of two wild-type Scn1a+/+ (#17 and #26) and two heterozygous Scn1a KT/+ (#21 and #23) mice, obtained from different parents are shown. No off-target effects were detected at three loci on mouse chromosome 2, namely, Scn3a, Scn7a, and Cd44. At each locus, the region highlighted in blue corresponds to the potential off-target site for the guide RNA (sgRNA72) and the red line denotes the PAM sequence. The Cas9 cut site is shown by the vertical double arrowhead line. Download Figure 1-1, DOCX file. [file enu-eN-NWR-0394-20-s04.docx]

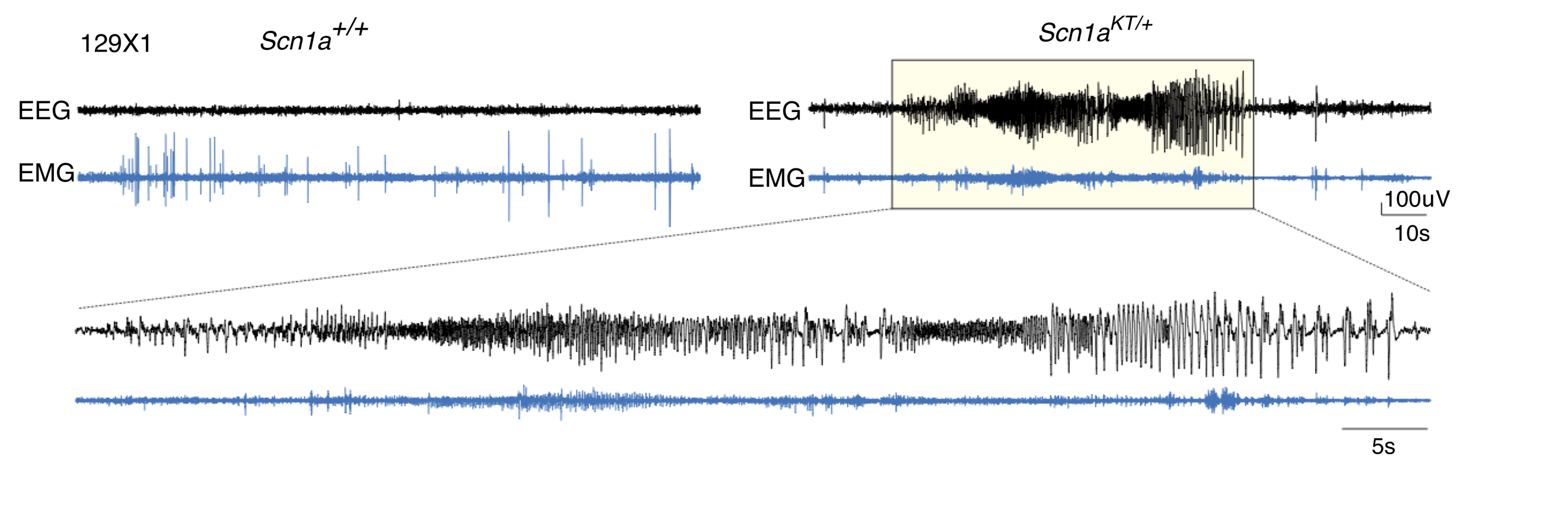


Extended Data Figure 3-1

Supplement: Extended Data Figure 3-1 — Representative electrographic traces from a four-month-old wild-type (Scn1a+/+) and a heterozygous (Scn1aKT/+) mouse on 129X1 strain. Left, Normal EEG and EMG traces from a wild-type mouse during baseline activity. Right, Example EEG and corresponding EMG recordings during a spontaneous seizure episode (shaded box) in the heterozygous mouse. The seizure episode, highlighted in the shaded box, shows a classic EEG waveform with high amplitude and high frequency polyspike discharges is expanded below. This animal experienced an average of 18.1 ± 5.3 seizures per day with a mean seizure duration of 38.9 ± 6.2 s. Download Figure 3-1, DOCX file. [file enu-eN-NWR-0394-20-s05.docx]

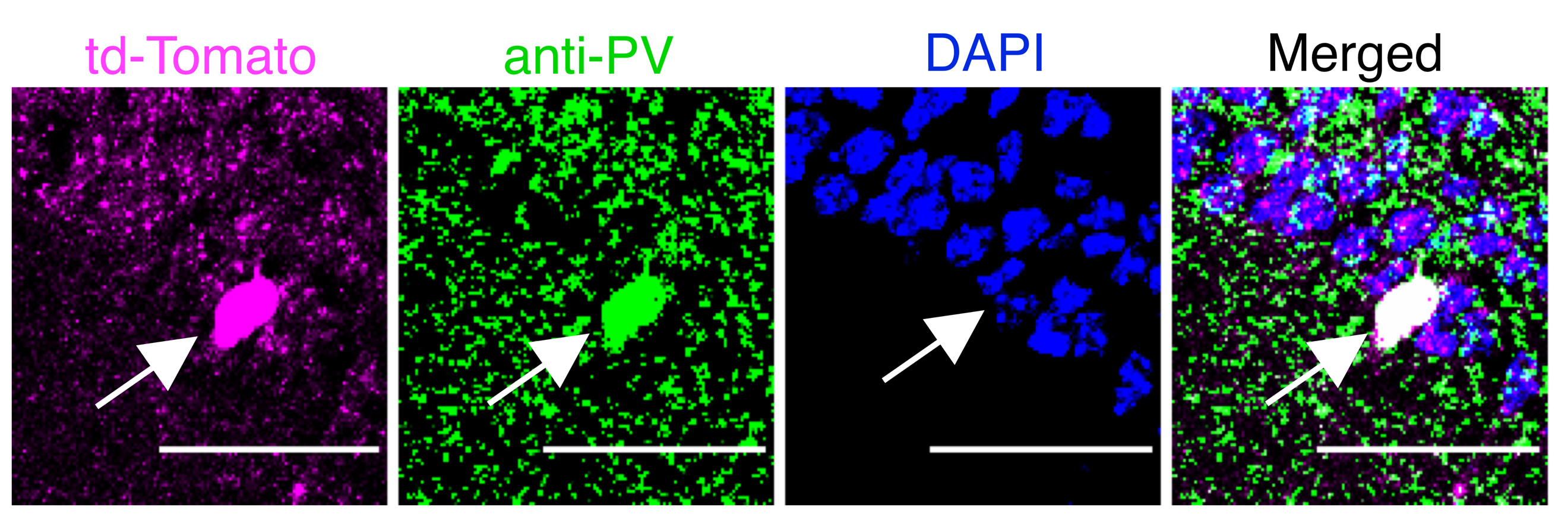


Extended Data Figure 4-1

Supplement: Extended Data Figure 4-1 — A representative PV interneuron (arrow) which was genetically labeled with td-Tomato (magenta) in Scn1aKT/+;PV-Cre;Ai14-tdTomato mice and co-immunostained with anti-PV antibody (green) and DAPI (blue). Right most panel shows the merged image. Scale bar: 50 μm. Download Figure 4-1, DOCX file. [file enu-eN-NWR-0394-20-s06.docx]
