# Supplementary material for: Interneuron Dysfunction in a New Mouse Model of SCN1A GEFS+
Source: eNeuro. 2021 Apr 8;8(2):ENEURO.0394-20.2021. doi: 10.1523/ENEURO.0394-20.2021 (PMC8174035; doi:10.1523/ENEURO.0394-20.2021)
Supplement: Extended Data Table 2-1 — Individual data points for (A) survivorship curve and (B) mean body weight of mice across postnatal age (days) in both strains, 129 and B6N. na, not applicable; d, dead. M1,2,3…, individual mouse under study. Download Table 2-1, DOCX file. [file enu-eN-NWR-0394-20-s03.docx]

| **Postnatal age of mice (Days)** | **Strain : 129**  **No. of mice (mice %)** | | | **Strain: B6N**  **No of mice (mice %)** | | |
| --- | --- | --- | --- | --- | --- | --- |
|  | ***Scn1a^KT/KT^*** | ***Scn1a^KT/+^*** | ***Scn1a^+/+^*** | ***Scn1a^KT/KT^*** | ***Scn1a^KT/^*** | ***Scn1a^+/+^*** |
| 0 | 8 (100) | 26 (100) | 12 (100) | 3 (100) | 12 (100) | 3 (100) |
| 1 | 8 (100) | 26 (100) | 12 (100) | 3 (100) | 12 (100) | 3 (100) |
| 2 | 8 (100) | 26 (100) | 12 (100) | 3 (100) | 12 (100) | 3 (100) |
| 3 | 8 (100) | 26 (100) | 12 (100) | 3 (100) | 12 (100) | 3 (100) |
| 4 | 8 (100) | 26 (100) | 12 (100) | 3 (100) | 12 (100) | 3 (100) |
| 5 | 8 (100) | 26 (100) | 12 (100) | 3 (100) | 12 (100) | 3 (100) |
| 6 | 8 (100) | 26 (100) | 12 (100) | 3 (100) | 12 (100) | 3 (100) |
| 7 | 8 (100) | 26 (100) | 12 (100) | 3 (100) | 12 (100) | 3 (100) |
| 8 | 8 (100) | 26 (100) | 12 (100) | 3 (100) | 12 (100) | 3 (100) |
| 9 | 8 (100) | 26 (100) | 12 (100) | 3 (100) | 12 (100) | 3 (100) |
| 10 | 8 (100) | 26 (100) | 12 (100) | 3 (100) | 12 (100) | 3 (100) |
| 11 | 8 (100) | 26 (100) | 12 (100) | 3 (100) | 12 (100) | 3 (100) |
| 12 | 8 (100) | 26 (100) | 12 (100) | 3 (100) | 12 (100) | 3 (100) |
| 13 | 8 (100) | 26 (100) | 12 (100) | 3 (100) | 12 (100) | 3 (100) |
| 14 | 8 (100) | 26 (100) | 12 (100) | 3 (100) | 12 (100) | 3 (100) |
| 15 | 8 (100) | 26 (100) | 12 (100) | 3 (100) | 12 (100) | 3 (100) |
| 16 | 8 (100) | 26 (100) | 12 (100) | 3 (100) | 12 (100) | 3 (100) |
| 17 | 8 (100) | 26 (100) | 12 (100) | 3 (100) | 12 (100) | 3 (100) |
| 18 | 6 (75) | 26 (100) | 12 (100) | 2 (66.67) | 12 (100) | 3 (100) |
| 19 | 6 (75) | 26 (100) | 12 (100) | 2 (66.67) | 12 (100) | 3 (100) |
| 20 | 5 (62.5) | 26 (100) | 12 (100) | 2 (66.67) | 12 (100) | 3 (100) |
| 21 | 2 (25) | 26 (100) | 12 (100) | 1 (33.33) | 12 (100) | 3 (100) |
| 22 | 1 (12.5) | 26 (100) | 12 (100) | 0 (0) | 12 (100) | 3 (100) |
| 23 | 1 (12.5) | 26 (100) | 12 (100) | na | 12 (100) | 3 (100) |
| 24 | 0 (0) | 26 (100) | 12 (100) | na | 12 (100) | 3 (100) |
| 25 | na | 26 (100) | 12 (100) | na | 12 (100) | 3 (100) |
| 26 | na | 26 (100) | 12 (100) | na | 12 (100) | 3 (100) |
| 27 | na | 26 (100) | 12 (100) | na | 12 (100) | 3 (100) |
| 28 | na | 26 (100) | 12 (100) | na | 12 (100) | 3 (100) |
| 29 | na | 26 (100) | 12 (100) | na | 12 (100) | 3 (100) |
| 30 | na | 26 (100) | 12 (100) | na | 12 (100) | 3 (100) |
| .  . | .  . | .  . | .  . | .  . | .  . | .  . |
| 180 | na | 26 (100) | 12 (100) | na | 12 (100) | 3 (100) |

Table A. Survivorship data

Table B. Body weight of mice (in gms) across postnatal age (Days)

| **Strain: 129** | | | | | | | | | | |
| --- | --- | --- | --- | --- | --- | --- | --- | --- | --- | --- |
| ***Scn1a^+/+^*** | **Day5** | **Day8** | **Day11** | **Day14** | **Day17** | **Day20** | **Day23** | **Day26** | **Day29** | **Day32** |
| M1 | 1.81 | 2.9 | 4.27 | 5.58 | 5.98 | 6.67 | 8.16 | 9.65 | 10.5 | 12.15 |
| M2 | 2.25 | 3.77 | 4.99 | 5.98 | 6.46 | 7.19 | 8.945 | 10.7 | 11.92 | 14.07 |
| M3 | 2.7 | 4.13 | 6.49 | 7.11 | 7.53 | 9.49 | 11.45 | 12.35 | 14.8 | 16.64 |
| M4 | 3.04 | 4.67 | 6.12 | 7.19 | 8.09 | 9.14 | 11 | 12.5 | 15.41 | 15.71 |
| M5 | 2.82 | 4.8 | 5.94 | 7.2 | 8.13 | 9.23 | 10.43 | 11.82 | 13.99 | 15.04 |
| M6 | 2.64 | 4.9 | 5.73 | 6.63 | 7.9 | 8.05 | 10.5 | 11.81 | 15.04 | 17.08 |
| M7 | 2.83 | 3.7 | 6.05 | 7.09 | 8.29 | 8.98 | 11.14 | 12.7 | 15.72 | 17.71 |
| M8 | 2.97 | 4.28 | 5.84 | 7.19 | 7.44 | 7.54 | 10.77 | 11.45 | 13.24 | 15.48 |
| M9 | 2.85 | 4.1 | 5.39 | 7.1 | 7.39 | 7.78 | 10.36 | 10.82 | 12.38 | 13.81 |
| M10 | 3.02 | 4.32 | 5.76 | 7.15 | 7.53 | 7.61 | 10.55 | 11.45 | 12.69 | 14.68 |
| M11 | 2.84 | 4.37 | 5.77 | 7 | 7.51 | 7.75 | 11.15 | 12.02 | 14.74 | 17.89 |
| M12 | 2.89 | 4.38 | 5.83 | 6.93 | 7.2 | 7.42 | 10.03 | 11.25 | 14.18 | 17.54 |
|  |  |  |  |  |  |  |  |  |  |  |
| ***Scn1a^KT/+^*** | **Day5** | **Day8** | **Day11** | **Day14** | **Day17** | **Day20** | **Day23** | **Day26** | **Day29** | **Day32** |
| M1 | 2.11 | 3.68 | 5.37 | 6.97 | 7.23 | 8.11 | 9.855 | 11.6 | 12.53 | 14.92 |
| M2 | 2.17 | 3.5 | 5.07 | 7.52 | 7.78 | 8.26 | 10.13 | 12 | 13.13 | 15.58 |
| M3 | 2.47 | 3.8 | 5.31 | 7.02 | 7.55 | 8.01 | 10.405 | 12.8 | 14.3 | 16.63 |
| M4 | 2.1 | 3.56 | 5.16 | 6.28 | 7.35 | 7.43 | 9.74 | 12.05 | 13.57 | 15.95 |
| M5 | 2.76 | 3.92 | 5.82 | 7.03 | 7.35 | 9.12 | 10.9 | 12.17 | 14.9 | 16.86 |
| M6 | 2.75 | 4.11 | 5.83 | 6.8 | 7.46 | 8.83 | 10.2 | 10.3 | 12.8 | 14.26 |
| M7 | 3.04 | 4.35 | 6.42 | 8.62 | 9.055 | 9.49 | 9.51 | 12.6 | 13.79 | 16.89 |
| M8 | 3.52 | 4.95 | 6.78 | 8.46 | 8.85 | 9.24 | 10.04 | 12.54 | 15.84 | 19.38 |
| M9 | 2.64 | 4.21 | 5.66 | 6.78 | 7.51 | 8.11 | 9.84 | 11.45 | 14.14 | 16.93 |
| M10 | 2.83 | 3.7 | 6.05 | 7.09 | 8.29 | 8.98 | 11.14 | 12.7 | 15.72 | 17.71 |
| M11 | 2.24 | 3.81 | 5.4 | 7.57 | 8.2 | 8.51 | 9.755 | 11 | 12.01 | 13.48 |
| M12 | 1.94 | 3.4 | 5.02 | 6.24 | 7.32 | 7.61 | 9.195 | 10.78 | 11.77 | 13.3 |
| M13 | 2.44 | 3.85 | 5.33 | 6.89 | 7.88 | 8.17 | 9.985 | 11.8 | 13.35 | 14.82 |
| M14 | 2.91 | 4.38 | 6.33 | 7.27 | 7.89 | 9.04 | 10.18 | 10.8 | 12.31 | 14.02 |
| M15 | 3.2 | 4.5 | 6.2 | 7.38 | 8.18 | 9.39 | 10.6 | 11.3 | 12.8 | 14.33 |
| M16 | 3.3 | 4.68 | 6.74 | 8.2 | 8.585 | 8.97 | 10.3 | 11.56 | 12.12 | 14.19 |
| M17 | 3.6 | 4.86 | 6.97 | 8.96 | 9.15 | 9.34 | 10.93 | 12.13 | 12.88 | 15.05 |
| M18 | 3.7 | 5.16 | 7.05 | 8.79 | 9.135 | 9.48 | 10.96 | 12.61 | 13.58 | 15.59 |
| M19 | 2.65 | 3.81 | 5.95 | 6.99 | 8.29 | 8.84 | 10.69 | 12.22 | 14.29 | 15.49 |
| M20 | 3.12 | 4.78 | 6.29 | 7.54 | 7.82 | 8.35 | 11.55 | 12.07 | 13.93 | 16.15 |
| M21 | 2.83 | 4.38 | 5.97 | 7.45 | 7.67 | 8.12 | 10.96 | 11.62 | 14.4 | 16.09 |
| M22 | 2.65 | 4.56 | 5.98 | 6.99 | 7.34 | 7.6 | 10.51 | 11.35 | 12.51 | 15 |
| M23 | 3.03 | 4.35 | 5.75 | 7 | 7.15 | 7.46 | 10.63 | 11.54 | 13.99 | 17.16 |
| M24 | 2.61 | 4.05 | 5.38 | 6.49 | 6.88 | 6.72 | 9.31 | 9.47 | 10.84 | 12.32 |
| M25 | 3.35 | 4.89 | 6.55 | 8.14 | 8.3 | 8.66 | 11.89 | 12.62 | 14.83 | 17.05 |
| M26 | 2.98 | 4.85 | 6.49 | 7.45 | 8.89 | 8.26 | 11.38 | 11.82 | 13.89 | 15.94 |
|  |  |  |  |  |  |  |  |  |  |  |
| ***Scn1a^KT/KT^*** | **Day5** | **Day8** | **Day11** | **Day14** | **Day17** | **Day20** | **Day23** | **Day26** | **Day29** | **Day32** |
| M1 | 2.35 | 4.07 | 5.35 | 6.18 | 6.57 | 6.81 | d |  |  |  |
| M2 | 2.4 | 4.01 | 5.33 | 6.44 | 7.63 | 7.27 | d |  |  |  |
| M3 | 2.5 | 4.05 | 5.5 | 6.52 | 7.06 | 7.1 | 7.2 |  |  |  |
| M4 | 2.61 | 4.01 | 6.3 | 6.52 | 6.56 | d | d |  |  |  |
| M5 | 3.2 | 4.41 | 6.36 | 8.06 | 7.82 | 7.58 | d |  |  |  |
| M6 | 3.61 | 5.04 | 6.65 | 8.45 | 8.405 | 8.36 | d |  |  |  |
| M7 | 3.19 | 4.77 | 6.41 | 7.77 | 7.27 | d |  |  |  |  |
| M8 | 2.95 | 4.24 | 5.31 | 6.33 | 7.27 | d |  |  |  |  |
|  |  |  |  |  |  |  |  |  |  |  |

| **Strain: B6N** | | | | | | | | | | | | | | |
| --- | --- | --- | --- | --- | --- | --- | --- | --- | --- | --- | --- | --- | --- | --- |
| ***Scn1a^+/+^*** | **Day5** | **Day8** | **Day9** | **Day12** | **Day13** | **Day 15** | **Day16** | **Day18** | **Day20** | **Day21** | **Day23** | **Day24** | **Day27** | **Day30** |
| M1 | - | - | 5.2 | - | 6.5 | - | 7.5 | - | 9.7 | - | 12 | - | 15 | 16.7 |
| M2 | 2.2 | 3.3 | 3.6 | 4.7 | 4.9 | 5.2 | 5.7 | 5.9 | 7 | 7.4 | 7.9 | 8.3 | 10.4 | 12 |
| M3 | 1.8 | 2.4 | 2.8 | 4.3 | 4.2 | 5 | 5.2 | 6.2 | 6.2 | 7.3 | 8.3 | 9.5 | 11.7 | 13.3 |
|  |  |  |  |  |  |  |  |  |  |  |  |  |  |  |
| ***Scn1a^KT/+^*** | **Day5** | **Day8** | **Day9** | **Day12** | **Day13** | **Day 15** | **Day16** | **Day18** | **Day20** | **Day21** | **Day23** | **Day24** | **Day27** | **Day30** |
| M1 | - | - | 4.5 | - | 6.1 | - | 7.1 | - | 8.7 | - | 10.4 | - | 14.2 | 16 |
| M2 | - | - | 5.2 | - | 6.6 | - | 7.6 | - | 9.3 | - | 11.1 | - | 14.2 | 15.8 |
| M3 | - | - | 4.8 | - | 6.5 | - | 7.3 | - | 9 | - | 10.6 | - | 13.7 | 16 |
| M4 | - | - | 4.9 | - | 6.2 | - | 7.7 | - | 9.2 | - | 10.7 | - | 12.6 | 14.4 |
| M5 | 2.7 | 4 | 4.3 | 5.1 | 5.2 | 5.7 | 6.7 | 6.4 | 7.3 | 8.3 | 9.5 | 9.7 | 12 | 13.8 |
| M6 | 2 | 3.2 | 3.6 | 4.9 | 5.3 | 5.4 | 5.3 | 5.9 | 6.8 | 7.4 | 8.5 | 8.6 | 11.3 | 13.1 |
| M7 | 2.3 | 3.5 | 3.5 | 4.2 | 4.5 | 5 | 5.2 | 5.5 | 6.5 | 6.7 | 7.4 | 7.7 | 9.5 | 12 |
| M8 | 1.7 | 2.3 | 2.9 | 4.2 | 4.5 | 5.3 | 5.6 | 6.2 | 6.3 | 7.1 | 8.3 | 9.6 | 11.1 | 12.7 |
| M9 | 1.7 | 2.2 | 2.7 | 4.1 | 4.3 | 5.1 | 5.3 | 6.1 | 5.9 | 6.5 | 7.7 | 8.7 | 11.4 | 12.5 |
| M10 | 2.4 | 3.9 | 3.7 | 4.6 | 4.6 | 5 | 5.2 | 5.5 | 6 | 6.6 | 7.1 | 8 | 11.7 | 13.8 |
| M11 | 2.7 | 4 | 4.4 | 5.4 | 5.7 | 5.9 | 6 | 5.9 | 7.7 | 7.9 | 9 | 9.5 | 12.5 | 14.2 |
| M12 | 2.4 | 3.7 | 4 | 4.9 | 5.4 | 5.8 | 6.5 | 6.3 | 7.4 | 8 | 9 | 9.6 | 12.9 | 14.9 |
| M13 | 1.4 | 2.2 | 3 | 4.2 | 4.7 | 5.4 | 6 | 6.9 | 6.9 | 7.7 | 8.7 | 9.9 | 12.2 | 14.8 |
|  |  |  |  |  |  |  |  |  |  |  |  |  |  |  |
| ***Scn1a^KT/KT^*** | **Day5** | **Day8** | **Day9** | **Day12** | **Day13** | **Day 15** | **Day16** | **Day18** | **Day20** | **Day21** | **Day23** | **Day24** | **Day27** | **Day30** |
| M1 | - | - | 4.7 | - | 6.5 | - | 7.4 | d |  |  |  |  |  |  |
| M2 | 2.1 | 3.3 | 3.5 | 4.4 | 4.8 | 5 | 5.5 | 5.7 | 4.5 | 4.7 | d |  |  |  |
| M3 | 2.4 | 3.6 | 3.9 | 4.4 | 4.6 | 4.6 | 4.7 | 4.8 | 4.6 | d |  |  |  |  |
|  |  |  |  |  |  |  |  |  |  |  |  |  |  |  |

Extended Data Table 2-1. Individual data points for (A) survivorship curve and (B) mean body weight of mice (in gms) across postnatal age (days) in both strains – 129 and B6N. na – not applicable, d – dead. M1,2,3… individual mouse under study.
